# Advances in Research of Adult Gliomas

**DOI:** 10.3390/ijms22020924

**Published:** 2021-01-18

**Authors:** Alina Finch, Georgios Solomou, Victoria Wykes, Ute Pohl, Chiara Bardella, Colin Watts

**Affiliations:** 1 Institute of Cancer Genomic Sciences, University of Birmingham, Birmingham B15 2TT, UK; A.Finch.1@bham.ac.uk (A.F.); w4r99@students.keele.ac.uk (G.S.); V.Wykes@bham.ac.uk (V.W.); 2 School of Medicine, Keele University, Staffordshire ST5 5NL, UK; 3 Department of Neurosurgery, University Hospital Birmingham, Birmingham B15 2WB, UK; 4 Department of Cellular Pathology, University Hospital Birmingham, Birmingham B15 2WB, UK; ute.pohl@nhs.net

**Keywords:** diffuse gliomas, experimental models, glioma heterogeneity, clinical trial

## Abstract

Diffuse gliomas are the most frequent brain tumours, representing 75% of all primary malignant brain tumours in adults. Because of their locally aggressive behaviour and the fact that they cannot be cured by current therapies, they represent one of the most devastating cancers. The present review summarises recent advances in our understanding of glioma development and progression by use of various in vitro and in vivo models, as well as more complex techniques including cultures of 3D organoids and organotypic slices. We discuss the progress that has been made in understanding glioma heterogeneity, alteration in gene expression and DNA methylation, as well as advances in various in silico models. Lastly current treatment options and future clinical trials, which aim to improve early diagnosis and disease monitoring, are also discussed.

## 1. Introduction

A brain tumour is formed when there is an abnormal clonal growth of cells within the brain. Brain tumours can be classified into malignant (cancerous) and benign (non-cancerous). Malignant tumours can be primary or secondary; the former originate from cells within the central nervous system (CNS), while the latter arise from cancer cells, which have metastasised from other tissues of the body to the brain via haematogenous spread. Two main cell types compose the CNS: neurons, which act as the communicators of the nervous system, and glia (or glial cells or neuroglia), which provide support and protection to neurons, maintain homeostasis and form myelin sheaths around them [1]. In the CNS, glial cells include oligodendrocytes, astrocytes, ependymal cells, and microglia. Tumours arising from glial cells or its precursors are called gliomas, and those which histologically appear similar to oligodendrocytes and astrocytes are defined as “diffuse glioma”; this term includes astrocytomas and oligodendrogliomas, graded as World Health Organization (WHO) grade II or III, and glioblastomas, assigned WHO grade IV.

Gliomas are the most frequent (75%) of all primary malignant brain tumours in adults [2]; because of their locally aggressive behaviour and the fact that they cannot be cured by current therapies, they are considered one of the most devastating cancers.

**Figure 1:**
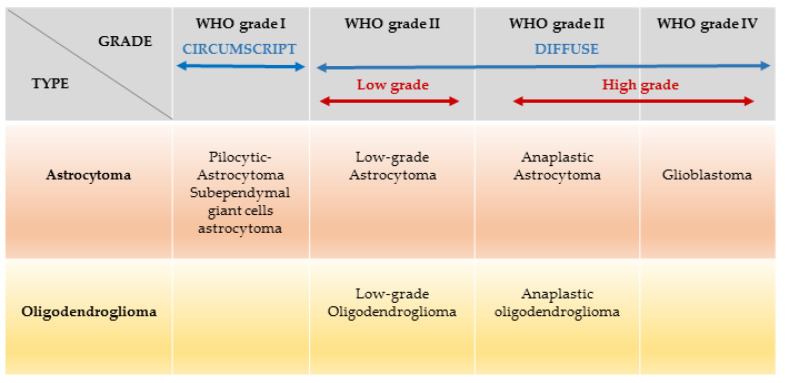
Gliomas classification based on WHO grades and cell types: the term diffuse glioma does not refer to circumscribed astrocytomas, which are benign tumours (e.g., Pilocytic astrocytoma, subependymal giant cell astrocytoma. Amended from Chemotherapy in glioma Walter Taal et al. CNS Oncol. 2015 May; 4(3): 179–192.

The revised 2016 WHO Classification of Tumours of the CNS divides gliomas into low grade glioma (LGG; WHO I-II) and high-grade glioma (HGG; WHO III-IV) based on integrated classic histological features and molecular biomarkers [2] Figure 1.

## 2. Epidemiology and Classification of Adult Gliomas

Of all adult primary brain tumours, glioblastoma (median age of 46.3 years at diagnosis [3]) is the most common (56.6%) and most aggressive for which there is no curative treatment [4]. Recent evidence suggests that there has been a significant increase in the incidence of glioblastoma from 1995 to 2015 more than doubling from 2.4 to 5.0 per 100,000 in the UK, whereas the rest of gliomas remained stable [5]. Further, this type of tumour is expected to rise dramatically in the U.S.A in the next 30 years, whereas the remaining gliomas will remain stable [6]. Factors postulated to contribute to this increase include an aging population, wider access to neuroimaging and exposure to ionising radiation [7]. Despite optimal surgery and oncological therapy, the glioblastoma survival rate remains significantly lower (median survival of 15 months [8]), compared to other glioma subtypes. The five-year survival rate for glioblastoma in the U.S.A is 6.8%, compared to 51.6% of diffuse astrocytoma, 30.2% of anaplastic astrocytoma, 82.7% oligodendroglioma, and 60.2% anaplastic oligodendroglioma [2]. Glioblastoma may develop rapidly without evidence of a less malignant precursor lesion (primary glioblastoma or ‘de novo’ glioblastoma), or through progression from a lower grade tumour (secondary glioblastoma). Low grade gliomas (astrocytoma, and oligodendrogliomas grade II) normally affect young adults (median age at diagnosis is 35 years for grade II astrocytoma and 34.8 years for grade II oligodendrogliomas [3]) with a survival average of approximately 7 years for patients affected by astrocytomas and more than 15 years for those who have developed oligodendrogliomas [9]. Although low grade glioma patients have better survival rates compared to patients with high grade glioma, all low grade gliomas are invasive and eventually progress to high grade glioma (grade III/IV) leading to death [9]. In an attempt to develop new therapeutic strategies, the scientific community has taken steps forward to understand the genetic mutations, demystify the cell of origin of these tumours and identify the role the immune environment plays in the complex interplay of tumour formation and evolution.

In 2007, the World Health Organisation (WHO), has classified brain tumours based on cell type and grading (grades I–IV), using histopathological criteria which refer to similarities with putative cells of origin and presumed levels of differentiation [10]. The plethora of recent advances made in genomic sciences, molecular pathology, immunology, and disease modelling, have increased the knowledge in brain tumour neurobiology, and define a new era in neuro-oncology. Consequently, in 2016, the WHO classification has undergone major reconstruction. Malignant tumours of the CNS are now classified not only based on histopathological findings, but also on specific molecular genetic parameters [11]. In 2007, histopathological classification of ‘diffuse gliomas’ comprised four grades: (i) grade I, referring to lesions with low proliferative potential, generally curable following surgical resection alone; (ii) grade II, referring to diffuse astrocytoma and oligodendroglioma, infiltrative lesions which, despite low proliferative activity, often recur and progress to higher grade; (iii) grade III, referring to anaplastic astrocytoma and anaplastic oligodendroglioma, which are rapidly growing tumours often extensive, which spread into nearby tissues; and (iv) grade IV, denoted glioblastoma, with the most aggressive histological and clinical features. The new classification instead combines histopathological and genotypic nomenclature, where the phenotypic name is followed by the genetic classification (i.e., diffuse astrocytoma, IDH mutant) [11]. Further, the WHO clarifies that in case of discordance between genotype and phenotype, the former overrides. The new notion that genomic characterisation trumps histopathological observation, allows us to slowly move towards a new diagnostic and therapeutic approach based on objective genotypic description.

In the WHO classification of 2016, “diffuse gliomas” (Grades II–IV) are classified in two groups based on a hallmark genetic feature, the mutational status of the isocitrate dehydrogenase genes (*IDH1* and *2*), wildtype (non-mutant) versus mutant (Table 1). IDH mutations have been found in nearly 80% of the lower-grade gliomas and secondary glioblastomas, and in around 10% of primary glioblastomas [12]. *IDH* mutations are thought to occur early on in the genesis of a glioma [13] with the most common heterozygote mutation being *IDH1* Arg 132 (R132H). The mutated IDH enzyme reduces α-ketoglutarate (α-KG) to D-2-hydroxyglutarate (D-2HG) [14]. D-2HG is thought to act as an oncometabolite, leading to profound cell modifications including histone and DNA hypermethylation, inhibition of cell differentiation and increased proliferation [15,16], possibly by competitive inhibition of α-KG-dependent dioxygenases [17].

90% of glioblastomas (grade IV), termed as primary or de novo, and roughly 30% of diffuse and anaplastic astrocytomas (grade II and III, respectively) contain the wildtype *IDH* gene [18,19]. Further, mutations within the core promoter of telomerase reverse transcriptase (*TERT*) can also be found within this group [20,21], resulting in an increased telomerase activity, an important step in the immortalization process [22]. After the publication of data from The Cancer Genome Atlas (TCGA), glioblastoma was shown to have specific tumour suppressors inactivated, such as TP53 and retinoblastoma (RB). Moreover, these data also indicated the presence of a pro-oncogenic pathway activation in glioblastomas, such as the dysregulation of the receptor tyrosine kinase (RTK), Ras and phosphatidyl inositol 3-kinase (PI3K) pathways, via activation of growth factor receptors (EGFR, PDGFRA) and through neurofibromin 1 (NF1) and phosphatase and tensin homolog (PTEN) deletion [23]. IDH-wildtype “diffuse” gliomas are associated with worse prognosis [10,24].

Seventy percent of diffuse and anaplastic astrocytomas (grades II-III), 100% of diffuse and anaplastic oligodendrogliomas, and 10% of glioblastomas [18,19] are IDH mutant. These tumours have two further sets of diagnostic and prognostic subgroups. The first group refers to diffuse and anaplastic oligodendrogliomas (grades II-III) based on the fact that 100% have a codeletion of chromosomal arms 1p and 19q [in short 1p19q] forming a non-balanced centromeric translocation t(1; 19) (q10; p10). This codeletion is associated with better outcomes and better response to alkylating chemotherapy [11,19,24,25,26]. Although 1p19q codeletion was discovered nearly two decades ago, very little is known regarding its functional impact in the neurobiology of oligodendrogliomas. To date, evidence exists to suggest that TERT promoter mutation is highly prevalent within mutants [27], leading to an increased TERT promoter transcriptional activity, and consequent telomerase activity, which ultimately determine a crucial progression to immortalization [22,28]. Further, whole exosome studies reported that the capicua transcriptional repressor gene (CIC), is frequently and specifically mutated in 1p19q co-deleted oligodendrogliomas [29,30].

The second group refers to the majority of grade II–III gliomas that carry mutations in the transcriptional regulators ATRX and TP53 but no 1p19q codeletion, encompassing the diffuse or anaplastic astrocytomas (II–III) and secondary glioblastomas (IV) [31]. Deciphering the specific role of *ATRX* mutations to the tumour neurobiology has not been established to date, although there is some evidence to suggest it has a role in cell cycle regulation, histone regulation, and chromatin remodelling [32].

A major achievement in the WHO classification of 2016 is the (subtotal) omission of the previously ill-defined diagnoses of oligoastrocytoma and anaplastic oligoastrocytoma. By using both genotype (i.e., *IDH* mutations and 1p19q codeletion status) and phenotype as diagnostic criteria, most (if not all) tumours of this group fall into the category of oligodendroglioma or astrocytoma [33,34,35]. Only very rare reports describe tumours as “true” oligoastrocytomas, consisting of histologically and genetically discrete astrocytic (*IDH*-mutant, *ATRX*-mutant, 1p/19q-intact) and oligodendroglial (*IDH*-mutant, *ATRX*-wildtype, and 1p/19q-codeleted) mixed populations of tumour cells [36,37].

Consequently, accordingly to the CNS WHO 2016 classification criteria, the oligoastrocytoma diagnosis needs to be used only when diagnostic molecular testing is not available, or in the rare real cases where a diagnosis based on histological and molecular genetic features has identified a mixed population of oligodendroglioma and astrocytoma cells within the tumours.

## 3. Experimental Models of Adult Glioma

In vitro and in vivo models of human gliomas have great potential not only to improve our understanding of glioma biology, but also to allow the development of novel therapeutic strategies. Current adult glioma models have strengths and weaknesses (Table 2). Ideally, a preclinical glioma model should reproduce the following requirements: a genetic background similar to that of human gliomas; genetic, epigenetic, and phenotypic intratumoural heterogeneity resembling human glioma; the complex interactions between malignant and non-transformed stroma cells (such as immune cells, fibroblasts, endothelial cells) that create the tumour microenvironment should be faithfully represented by the model; the model should be reproducible and stable over time [38].

### 3.1. Cell Lines

In general, cell lines derived from human tumours are a common method for studying individual hallmarks of cancer such as sustained proliferative signaling, activation of invasive and metastatic programs, or resistance to apoptosis. In particular, primary or immortalized cell lines derived from human brain tumours with different grade of malignancy and various genetic complexity represent a common and valuable tool for studying oncogenic cellular and molecular mechanisms involved in glioma formation, growth, and progression, in a reproducible and controlled fashion. These cells can also be subsequently propagated subcutaneously or orthotopically as xenograft in mouse models. Additionally, cell cultures derived from human tumours can be also used to track the potential of multiple drugs at once, in rapid and repeatable pre-screening assays, before further testing in appropriate preclinical models. Common cell lines derived from high grade glioblastomas, such as U-87MG, LN-18, and LN-229 have provided a useful tool in studying many different drug approaches, such as how caffeine can sensitize glioblastoma cells to the effects of temozolomide (TMZ), or how tumour cells overcome contact inhibition [39,40]. However, it has been documented that in vitro long-term culture of cells in the presence of serum represents a selection pressure. Over time, this may result in genetic aberrations which cause changes in the gene expression and phenotype of the cancer cells [41,42,43,44]. This may impact responses to drugs and other stimuli, resulting in false positive or negative results, e.g., potentially beneficial drugs may be discarded in the early stages of research and conversely, drugs that are of little or no clinical benefit may be carried through to animal models and even clinical trials. To minimize the genetic alterations occurring during monolayer cell line culture of tissue derived from brain tumours, new culture methods have been developed, such as the neurosphere cultures. To form neurospheres, freshly resected human glioma cells are grown in a serum-free medium containing basic fibroblast growth factor (bFGF), epidermal growth factor (EGF), and the neuronal cell culture supplement B-27 [45,46,47]. These culture conditions have several advantages compared to monolayer cultures, such as the selection and expansion of glioma stem-like cells from explanted gliomas [48,49,50]. Indeed glioma stem-like cells more closely retain the phenotype and genotype of the primary tumour compared to the serum-cultured cell lines [51]. However, it is not possible to develop neurosphere cultures from all human gliomas, and successful culture depends on the histological grade and genetic background of the tumour analysed [48].

### 3.2. Cerebral Organoids

The use of cerebral organoids is becoming a more attractive approach when investigating human glioma biology. They can be derived from human pluripotent stem cells including human induced pluripotent stem cells (iPSC) and human embryonic stem cells (ESC) [52]. Cerebral organoids represent a miniature organ grown in vitro, which contains several types of neural cells and have anatomical features that resemble a simplified mammalian brain.

Cerebral organoids are a more complex and physiological system to study brain cancer compared to other in vitro and in vivo models. Cell lines and patient-derived tumour xenografts (PDTXs) used to study tumorigenesis have their limitations: genetic changes acquired during the in vitro cultures and two dimensional (2D) grown might make the findings obtained by using these methods unreliable; PDTXs models are expensive and require long term studies [53]. Cerebral organoids grow in suspension in a 3D culture system, which allow them to more faithfully represent the behaviors of cells seen in vivo such as invasion, because the suspended cells can grow in any direction, unconstrained by culture dishes [54]. This makes cerebral organoids particularly valuable for brain cancer research. Glioma stem cells isolated from human brain cancer [55], can also be cultured in a 3D extracellular matrix such as Matrigel [56], to form tumour spheres. However, tumour spheres embedded in Matrigel, or equivalent, consist of only the cancer stem cell population, whereas cerebral organoids follow a differentiation pathway that allow them to exhibit multiple cell types and patterning resembling an actual brain. This gives organoids a further advantage over tumour-derived cancer stem cells.

Recently, cerebral organoids derived from human ES cells following the protocol first described by Lancaster [57] have been used to model the early events in gliomagenesis [58]. By targeting the HRas^G12V^-IRES-tdTomato construct into the *TP53* locus by homologous recombination using CRISPR-Cas9 technology, Ogawa et al. were able to cause simultaneous disruption of TP53 and gain of function of HRas proteins, generating tumour formation. Organoids positive for tdTomato reporter were shown to exhibit a greater proportion of proliferative cells positive for the marker Ki67, when compared to controls represented by untransduced organoids. Flow cytometry showed that at 8 weeks post-electroporation, 5.7% of the organoid culture was composed of tumourigenic cells (tdTomato positive), which greatly increased to 86.8% at 16 weeks post-electroporation. Tumourigenic cerebral organoids also demonstrate the ability to proliferate and invade otherwise healthy organoid [58]. This study was able to very closely mimic human GBM formation and invasion by using healthy organoid tissue, which has been genetically manipulated. This research revealed insights into the mechanisms employed during GBM invasion to the surrounding tissue and paves the way for future drug studies to combat invasive behavior.

Other studies have employed similar methods to study the invasive behaviour of multiple patient-derived GBM samples. Krieger et al. describe their use of human iPSC-derived cerebral organoids as scaffolds for the study of growth and invasion of GBM samples [59]. The fluorescently labelled GBM samples were mixed with healthy organoids and allowed to grow for 3 days. Following this, there was an extensive invasion from all GBM samples tested. These samples were in fact forming membrane protrusions (microtubes) from individual tumour cells, that connected with one another over varying distances, thus creating a network of GBM cell connections. This mixed healthy-GBM organoid model provides insight into the heterogeneity between different patient-derived tumours and differences in invasive behaviours which opens opportunities for personalized therapies.

Recently, Krieger et al. have investigated differentially expressed genes in GBM cultures in combination with/without normal organoid cultures derived from iPSC. By using single cell RNA-sequencing (scRNA-seq), they identify a subset of genes that were differentially expressed in patient samples that had been co-cultured with healthy organoids compared to patient samples with no co-culture or healthy organoids cultures alone. This gene set was upregulated across all GBM samples that had been co-cultured and included: Gap Junction Protein Alpha 1 (*GJA1)* encoding connexin-43, which allows gap junctions to form between GBM microtubes; Collagen Type IV Alpha 5 Chain (*COL4A5*) which encodes for a component of the extracellular matrix, and Glypican 3 (*GPC3)*, an activator of the Wnt signaling pathway previously associated with invasiveness. This data demonstrate how co-culture of patient-derived GBMs with iPSC-derived cerebral organoids provides the cellular interactions necessary to induce a change in the transcriptome towards invasion. This mixed-organoid model and scRNA-seq also describes the heterogeneity between different patients’ tumours and provides a way to test the individual tumour’s susceptibility to targeted drug therapies on a clinically relevant time scale.

So far, cerebral organoids lack a realistic microenvironment composed by vessels and immune cells. Nevertheless, these models might provide a unique opportunities for studying mechanisms of gliomagenesis, screening drug candidates and designing personalized therapeutic treatment for people with brain cancer.

### 3.3. Organotypic Brain Slice Cultures

Brain slices cultured in vitro present an excellent opportunity to study the interactions of glioma cells with the brain microenvironment and in particular to study mechanisms of tumour cells infiltration and migration [60,61,62]. In these models, glioma cells are microinjected ex vivo orthotopically into mouse brain slices, which can be prepared from young or adult rodents and maintained in cultures for weeks. Brain cultures retain their three-dimensional organization with the preservation of cytoarchitecture, cell function and vascular network, allowing glioma cells to interact directly with other brain cell types including neurons, astrocytes, oligodendrocytes, and the brain-specific extracellular matrix. Recently, Marques-Torrejon et al. implanted adult mouse coronal brain slice cultures with either GFP-expressing patient-derived glioma stem cells or GFP-expressing mouse glioblastoma-initiating cells. Both cell types engrafted into the cultures and could be maintained for up to three weeks. Engrafted cells showed distinct responses to diverse microenvironments in the brain tissue, and cells injected within the neural stem cell niche even responded to endothelial niche signals. However, after 3 weeks, the culture started losing its structure, indicating that organotypic brain slice cultures may present some limitations in pre-clinical drug studies longer than this period [63]. In another study Eisemann et al. used organotypic cultures to investigate the invasive nature of glioma by implanting tumour spheroids generated from either mouse or human cell lines. They found that genetic alterations to the donor mouse brain such as knockout of cell surface proteins led to a reduction in tumour cell invasion. This ex vivo assay demonstrates that genetically altered mice can be combined with organotypic culture to show which cell surface markers can be utilized by the glioma cells to drive their invasion [64]. Other studies have demonstrated the suitability of brain slice cultures to investigate the response of gliomas cells to anticancer drugs. Recently, Minami and co-authors used brain slice cultures for testing the individual pharmacological effects of cisplatin, TMZ and paclitaxel on a model of malignant glioma. This model was generated by injecting adult mice with *Inka/Arf^−/−^* neural stem cells transduced with the oncogene *HRasV12*. The drug screening performed showed that cisplatin was able to significantly reduce tumour size, TMZ inhibited tumour cell proliferation, while paclitaxel was shown to cause mitotic slippage of tumour cells [62]. Brain explant cultures permit the study of pharmacological responses of glioma in the microenvironment of their tissue of origin. However, this also means that drug screens performed on these models do not account for the changes in drug concentration within the brain due to the effect of the blood–brain barrier (BBB). In fact, the BBB limits the delivery of drugs to brain tumours, making it difficult to understand whether a lack of drug efficacy is caused by an absence of pharmacological action or due to hindered delivery [62]. Increasing the permeability of the BBB could be an alternative route for increasing the efficacy of drugs to the brain. However brain slice culture systems, by enabling compounds to directly access brain tissue from the culture medium, represent an advantage for assessing the action of drugs that do not cross the BBB, such as paclitaxel [62].

### 3.4. In Vivo Models of Human Gliomas

Mouse models allow the study of tumourigenesis in the context of a physiological environment, in which the tumour encounters consistent blood flow and nutrients, multiple cell types, and, usually, an intact immune system. All these factors combined provide a more dynamic and physiologically accurate system when compared to isolated cell lines that grow in a much less complex environment [65].

There are multiple methods to obtain mouse models of gliomas, including (1) genetically engineered mouse models (GEMMs) which involve altering specific genes known to influence glioma formation and progression and (2) xenograft models which utilize patient-derived cells/tumour fragments. Xenograft models are generated using either glioma cell lines or patient tumour samples cultured in vitro and later inserted directly into the mouse brain to induce tumour formation, or by injecting small fragments of human gliomas directly into the brain, thus bypassing the need for culturing the samples, which could lead to the loss/acquisition of genetic alterations due to adaptation of tumour cells to culture conditions [66].

### 3.5. Genetically Engineering Mouse Models of Glioma

In genetically engineered mouse models (GEMMs), specific genetic alterations identified in human tumours are introduced in the germline (knock-out, knock-in, transgenic models) or in the somatic cells (via viral-mediated gene delivery) of a mouse model to generate a de novo tumour. In these models, the genetic alterations of interest are targeted to a specific cellular population within the mouse tissue/organ, and to a specific developmental stage of the animal (in embryonic, postnatal or adult organs). GEMMs represent an important experimental tool to investigate the cellular and molecular events responsible for tumour initiation and progression, and to determine the minimum genetic alterations necessary for malignant transformation. Another important advantage represented by GEMM is that they have been established in immunocompetent animals, highlighting the importance of the microenvironment in tumour biology. GEMMs have been used for testing biomarker expression, drug delivery methods and new therapeutic strategies, and many studies using GEMMs have been successfully translated into clinical trials.

There are numerous glioma GEMMs some of which are reviewed here (for a comprehensive review see [43,67]).

A model of astrocytoma was developed by Reilly et al. by crossing two mouse strains, Nf1^+/−^ and Trp53^+/−^ to produce a double heterozygous mouse. This model produced mostly low-grade diffuse astrocytoma (WHO grade II), which in some cases progressed to a grade IV glioblastoma [68]. There have been many other models developed of high-grade astrocytoma that rely on genetic alterations to *Ras*, *EGFR*, and *Akt* among others [69]. Some models such as that generated by Marumoto and coauthors use lentiviral vectors as a means of transmitting the required genetic alteration into mouse brain. Lentiviruses were stereotaxically injected into the cortex, subventricular zone and hippocampus and lead to the generation of high grade astrocytomas (GBMs) [70]. GEMMs of oligodendroglioma of varying grades have also been developed. Ding and co-authors produced an oligodendroglioma model by crossing mice expressing an overactive form of EGFR (EGFRvIII), with mice carrying the Ha-RasV12 mutant, both GFAP promoter-driven [71]. These double mutant mice generated brain tumours that closely resembled the histology of oligodendroglioma, immunonegative for both Nestin and GFAP. Some EGFRvIII/Ha-RasV12 double mutant mice also demonstrated histological features of astrocytoma.

Although GEMMs are useful tools in understanding how acquisition of mutation(s) cause tumour formation, they also present some limitations: the genetic alterations generated are limited to a number of genes, thus they cannot fully represent the genetic complexity and heterogeneity that is often seen in resected human glioma samples. Moreover, their development is normally costly and time consuming.

### 3.6. Xenograft Models

In a patient derived xenograft (PDX) model, human gliomas in the form of tumour cell models or en bloc biopsies are injected or transplanted into an immunodeficient mouse to prevent rejection of the transplant from the host. Several strains of mice with different levels of inhibition of the immune system can be used in these studies: athymic nude mice, in which T-cells populations are greatly reduced; severely compromised immune deficient (SCID) mice, in which both B and T cells are lacking; NOD-SCID mice, in which there is an impaired development of B and T lymphocytes and natural killer (NK) cells; and recombination-activating gene 2 (Rag2)-knockout mice, in which both the maturation of both B- and T-cells is impaired. In these mice, the tumour sample can be implanted heterotopically, into an area of the mouse unrelated to the original tumour site (generally subcutaneously) or orthotopically, into the site of origin of the tumour (i.e., intracerebrally for brain tumours). Tumours generated in xenograft models grow relatively quickly and retain the cellular and molecular characteristics of the patient’s tumour. Preservation of the intra-tumoural genetic heterogeneity allows an accurate representation of the sample of origin. Furthermore, PDX have the advantage of developing an in vivo vascular supply. For these reasons, they represent a useful clinical model to test the response of tumour cells to therapy, albeit for a limited number of in vivo passages in the mouse. After which a selective pressure for propagation starts that causes specific genetic and epigenetic signature changes in the cells, making the generated tumours less representative of the original tumour from the patient [72].

In a heterotopic xenograft model, glioma cells are injected in a tissue where they will not experience the same stimuli and cell-cell interactions present in the microenvironment of the tissue of origin. Brain tumour samples will also lack input from brain-specific immune factors, such as microglia and macrophages, which can both affect and be effected by the tumour cells [73,74]. To reduce this limitation, stroma from the human tumour microenvironment can be included in the xenograft [75]. Alternatively, orthotopic xenografts represent a better experimental choice. In the orthotopic xenograft models of brain cancer, the tumour sample is injected stereotaxically into the mouse brain so that the effect of the cancer cells on its microenvironment can be at least in part measured. Intracranial models also allow for the testing of the ability of new drugs to permeate the blood–brain barrier and still be able to maintain the required drug dose within the brain tissue [76]. In these models to monitor tumour growth and responses to drug therapy imaging techniques are required, thus sometimes glioma samples are labelled with luciferase prior to intracranial injection [77].

Recently, a particular xenograft model using patient-derived glioblastoma cells was generated by implanting cells via intravitreal injection into the mouse eye [78]. This model allowed for the generation of tumours after approximately just 2 weeks, which consistently progressed to definitive masses by 4 weeks. This PDX model allows for drug screens to begin just 6 weeks following the initial surgery and would allow for a fast turnaround time regarding potentially beneficial, additional treatments. The 6-week window required for generating these patient-specific PDX models is shorter than the average time between the patient’s initial surgery and tumour recurrence, thus this provides the potential to delay or even prevent recurrence of the disease [78].

### 3.7. Xenograft in ‘Humanized’ Mice

Xenograft models using human tumour samples can provide an excellent insight into human glioma biology, however these models rely on immunodeficient murine hosts. Given the more recent interest in the role of the immune system in cancer progression, these xenograft models present some limitations, as they cannot fully recapitulate the ‘true’ microenvironment in which immune and cancer cells interact.

In ‘humanized’ NOD/SCID mice models a functional human immune system is generated, through engraftment with human primary hematopoietic stem cells (HSCs) typically derived from human foetal tissues, umbilical cord blood, or peripheral blood mononuclear cells (PBMCs). To facilitate engraftment, the human leucocyte antigen (HLA) of HSCs and PBMCs should be matched to the tissue being xenotransplanted [79]. Human glioma tumour is then grafted into the ‘humanized’ NOD/SCID mice model. However, currently, no single ‘humanized’ mice model provides the full spectrum of the human immune system, as these models often show poor B cell maturation [80], and lack the HLA class I and class II selecting elements for shaping the T cell repertoire [81]. Unfortunately, these mice can also develop graft versus host disease (GVHD) against the xenografted tissue. GVHD is a severe immune reaction to xenografted tissue and usually occurs because the xenografted tissue does not match that of the recipient [82]. The incidence of GVHD was especially pronounced in mice developed using the PMBC-transplant model. This may be overcome by using bone marrow from the same patient supplying the tissue to be xenografted, although this would be difficult to implement on a large scale [79,83].

Recently, studies of glioma samples transplanted into mice with intact immune systems have started to emerge. Semenkow et al. provided evidence of an immunocompetent mouse model that was able to more faithfully resemble the pathophysiology seen clinically in patients with GBMs, compared to transplants grown in immunodeficient hosts [84]. To facilitate the engraftment of glioma samples into immunocompetent mice, transient pharmacological blockade of T-cells activation was obtained by using MR1, an antibody against murine CD154 present on the surface of T-cells and abatacept, an antibody against CD80/CD86, that prevents the antigen-presenting cells from delivering the first T-cell stimulatory signal. Four treatments with both MR1 and abatacept were sufficient to allow xenograft growth in the newly termed “immunotolerant mice” comparable to that of the standard immunodeficient mice [84]. Magnetic resonance imaging of these immunotolerant mice demonstrated increased tumour contrast-enhancement compared to the immunodeficient mice [85,86]. This is indicative of a higher blood–brain-barrier permeability commonly present in high grade gliomas. Immunotolerant mice also demonstrate increased microglia activation, and both T-cell and leukocyte infiltration, demonstrating the presence of both the innate and adaptive immune system. Taken together these results are a promising insight into a novel mouse model with a functional immune system. This could pave the way for more clinically relevant future drug studies involving immunotherapy and cancer vaccines as well as allowing for a greater and more in depth understanding of the complex interactions between cancer evolution and the host immune system.

## 4. Intratumour Heterogeneity and Tumour Evolution of Adult Gliomas

### 4.1. Intra-Tumour Heterogeneity Using Novel Tissue Sampling

Intra-tumour heterogeneity describes a phenomenon in which different cells within the same tumour acquire distinct epigenetic and genomic alterations. This allows development of dissimilar morphological and phenotypic features and consequent divergent development from the progenitor cancer cell population. Intra-tumour heterogeneity has been proposed to be one of the major obstacles for conventional and targeted therapies to maintain remission [87,88,89,90]. Diffuse gliomas and especially glioblastoma, are known to exhibit intra-tumour heterogeneity (when the phenomenon occurs within the same tumour) and also inter-tumour heterogeneity (when it occurs between different tumours) [91].

Previously, intra-tumour heterogeneity was demonstrated at the level of *EGFR*, *PDGFRA*, and *MET* using fluorescent in situ hybridisation (FISH) [92]. Sottoriva A. et al., showed for the first time that fluorescent guided objective multiple tissue sampling within the same patient is paramount for interrogating heterogeneity within the same cancer sample, in this case glioblastoma [91]. This was based on the fact that a single biopsy is unlikely to present the full set of mutations, leading to bias of reported gene expression [90]. Thirty-eight tissue samples from 9 patients were profiled for genome-wide DNA somatic copy number, showing consistent heterogeneous putative driver mutations, such as copy number gain/amplification of the *PDGFRA*, *MDM4*, and *AKT3* loci, and deletion of the genomic locus containing *PTEN*. Furthermore, each tumour copy number alternations (CNA), were classified as ‘common’, ‘shared’, and ‘unique’, i.e., all tumour fragments, more than one but not all fragments, and only one fragment, having CNA respectively. The vast majority of CNAs (31/38), showed ‘shared’ and ‘unique’ alterations. It was deduced that ‘common’ aberrations should have occurred earlier in development, as all tumour fragments (four fragments per tumour) were extracted from the same tumour mass. While ‘unique’ and ‘shared’ alternations found in a subset of tumour cells should have occurred later on in the development. Therefore, it was implied that taking samples from multiple sites within the tumour, enables both spatial and temporal interrogation of mutations. ‘Common’ CNAs were found in chromosomes 7 and 10, of which the most frequent putative glioblastoma drivers, such as *EGFR*, *CDK6*, *MET*, and *PTEN*, are located. Using microarrays and hierarchical clustering, it was also found that distinct glioblastoma subtypes are present within the same tumour. By measuring the mitotic distance between cells within a fragment, they also demonstrated that different subclones exist within each fragment, and also that distinct cell lineages are present, illustrating a complex and dynamic hierarchy. The authors concluded that, the founder clone initially displayed amplification/gain of *EGFR*, *CDK6*, and *MET*, and loss/deletion of *CDKN2A/B*, *PTEN*, and *PARK2*. Later clones displayed altered gene expression and loss of chromosomes. The variation in gene expression in tumour development suggests that a single therapy is highly unlikely to be effective for each subclone, meaning that while it might be effective for one clone, the rest of the tumour will be able to survive by either having inherent resistance [93] or acquiring resistance after treatment.

### 4.2. Complex Environmental Factors Shaping Dynamic Intratumour Gene Expressions

A recent study by Patel et al., used scRNAseq to measure intra-tumour heterogeneity in patients affected by primary glioblastomas. Six thousand genes were analysed from 430 cells isolated from each tumour; this constitutes adequate coverage to characterise heterogeneous gene expression and delineate correlation between transcriptional and functional expression [94]. Extensive intra-tumoural heterogeneity at the transcriptional level was observed, involving multiple cellular properties, such as oncogenic signalling, proliferation, complement/immune response, and hypoxia. Although cells were grouped by tumour of origin, transcriptional expression was not unique to the same tumour, meaning that equal genes were expressed from cells across different tumours originating from different patients. Variability was noted in the expression and/or mutational status of important signalling molecules, such as the splicing patterns of receptor tyrosine kinases (RTKs), *EGFR* truncations and in frame deletions (EGFRvIII). Additionally, using hierarchical clustering and principle component analysis four meta signatures (immune, cell cycle, hypoxia, and oligodendroglioma origin) were comprised of clusters, which vary amongst the whole set of cells, enriched by genes related to each meta-signature. Quiescence markers were found to be significantly higher in non-cycling tumour cells. Clustering of genes anti-correlated to the cell cycle signature, 9/12 were within the hypoxia meta-signature. Reordering of the cells by hypoxic module score, showed clear gradients for two clusters (MGH28/31), implying that hypoxia as a factor of tumour microenvironment can play a role in gene expression. This finding emphasised previous findings reporting tumours containing niches with phenotypic changes including quiescence and self-renewal [95] and adaptation to hypoxia [96]. Finally, Patel et al. adopted the TCGA classification scheme to distinguish glioblastoma subtypes—i.e., proneural, neural, classical, and mesenchymal [94]. They identified that all five tumours contained cells originating from a common proneural subtype regardless of the overall dominant subtype within the tumour, in agreement of the findings of Sottoriva A. et al. [91]. Moreover, they confirmed the presence of multiple subtypes within the same tumour. Interestingly, they revealed that cells can simultaneously score highly for two subtypes, meaning ‘hybrid’ states are possible, reflecting aberrant developmental programmes. Perhaps the most clinically relevant outcome of this study was the notion that increasing tumour subtype heterogeneity is linked to worse prognosis. This landmark study highlights that within a single tumour, cells can be found with divergent, highly complex and heterogeneous gene expressions and are subject to a changing surrounding environment.

## 5. Tumour Microenvironment Variations in Tumour Type, Subtypes, and Type of Treatment

Failure of clinical trials to show survival benefit for “diffuse glioma” is well known [97,98]. The complex interplay between cancer cells and the dynamic tumour microenvironment (TME) is a hallmark “promoter” of tumour gene expression and transcriptomic adaptability, which affects its heterogeneity and evolution [99]. Understanding the key drivers of gene alteration and expression, and how gene expression relates to cellular phenotypes, will allow us to develop rational therapeutic interventions to change the fate of intra-tumour evolution and thus change drug resistance patterns.

In a recent study, the critical differences in gene expression between IDH mutant astrocytomas (A) versus oligodendrogliomas (O) were deciphered, by combining 9879 single cell RNA-seq profiles from 10 IDH-A tumours, 4347 single cell RNA-seq profiles from 6 IDH-O tumours, and 165 TCGA bulk RNA profiles [100]. Differences in bulk expression profiles between IDH-A and IDH-O were found to be mainly defined by genetic mutations and TME composition, but not by distinct expression programs of glial lineages in the malignant cells. Interestingly, IDH-A and IDH-O exhibited different TME composition, and in particular the abundance of microglia/macrophage cells.

A greater macrophage-like expression was found in tumours of higher histopathological grades (II vs. III vs. IV) and this was correlated with increased endothelial cell signature. TME composition might be partly influenced by tumour cell gene expression, i.e., *TP53* mutated in astrocytomas has been shown to influence the nuclear factor kappa beta *NF-kβ* immunoregulatory pathway [101]. In contrast to the difference in TME composition, both IDH-A and IDH-O showed the same developmental hierarchy, suggesting a common progenitor for all IDH mutant gliomas with NSC/NPC-like program. This suggests that new immunotherapy strategies targeting glioma phenotypes may represent a novel approach to treatment.

The TME plays an important role in gliomagenesis and may influence the tumour evolutionary process. Unsupervised transcriptome analysis of the TCGA data revealed four clusters of glioblastoma subtypes: classical (CL), mesenchymal (MES), neural (NE), and proneural (PN), each linked to specific genomic alternations [102]. MES is reported to have worse prognosis [100,103,104]. Switching from PN subtype to MES subtype has been discussed as one of the potential factors leading to treatment resistance in GBM recurrence [105,106]. Tumour microglia or tumour associated macrophages have been implicated to be regulating the PN to MES transition via the NF-kβ activation providing growth factors mediated proliferative signals [105,107,108]. This pathway might have the potential to be therapeutically targeted [94,109].

Tumour-associated macrophages (TAM) and microglia are innate immune cells that can make up to half of the tumour mass in glioblastoma. Brain TAM and microglia retain plasticity and exist in a spectrum of different cellular states classified according to their environment: M1 proinflammatory and M2 cytoprotective [110]. During the M1 phenotype they release chemical mediators such as nitric oxide (NO), reactive oxygen species (ROS), and pro-inflammatory cytokines. The M2 phenotype release anti-inflammatory cytokines such as: IL-4, IL-10, and TGF-β. It has been found that M2 glioma-associated microglia and macrophages were correlated with the grade of glioma [111]. Further, microglia (possible M2) were the main cell responsible for the IL-10 expression in malignant gliomas [112,113]. 87% of grade III and IV, and only 4% of grade II expressed were positively correlated to IL-10 mRNA levels, while elevated levels of IL-10 were found in 11% of LGGs and 63.6% of HGGs [112]. The M2 microglia/macrophage gene signature was also shown to have greater association with the MES subtype (13%) versus the PN (5%) [107,114,115]. The fact that the M2 microglia phenotype was found to be increased in higher grades of glioma and MES subtype, might suggest that they can be potential candidates for therapeutic interventions. Interestingly Neurofibromatosis type 1 (NF1) deficient MES glioblastoma cells were shown to have an increase in M2 macrophage gene signature [115], suggesting that NF1 deactivation may promote macrophage/microglia recruitment [116].

The type of oncological treatment can also be affected by the TME. It has been postulated that TMZ treatment can induce aberrant mutations [117,118]. The new hypermutations may generate new antigens that can be recognized by CD8^+^ T cells [119]. CD8^+^ T cells were found to be significantly increased at recurrence in comparison with their primary tumours [115]. Hypermutations after TMZ treatment may lead to a more immunological reactive microenvironment, which can be subject to immune checkpoint inhibitors [120]. Further, short term relapse of glioblastoma was shown to have higher predicted presence of M2 macrophages phenotype. M2 macrophages have been speculated to play a role in resistance to radiotherapy in the past [121]. Therefore, macrophage-targeting therapy might be an option after radiotherapy cycle has be concluded [122,123].

## 6. Computational and Mathematical Modelling of Glioma

### 6.1. Modelling Parameters of Glioma Invasion and Progression

Glioblastoma metastasis outside the brain are rare [124], while this tumour diffusely infiltrates brain regions along blood vessels and white matter tracts. In fact GBM typically recurs within 1–2 cm of the primary tumour resection border, and generally the main cause of treatment failure and tumour recurrence is diffuse invasion of GBM cells into the surrounding brain tissue [125]. Migration and invasion of glioma cells into the adjacent tissue represent an area of great interest, and whilst the two mechanisms are complimentary to each other, they are separate processes where migration is the detachment and movement of glioma cells away from the main tumour mass, whilst invasion is the expansion of glioma cells into neighbouring healthy tissue without detaching from the tumour mass [126]. Because of the highly invasive nature of glioma, it has become increasingly important to be able to model and predict this behaviour in silico [127].

Invasion of glioma cells into normal tissue is a complex process, which is determined by complex interactions between glioma cells and the extracellular microenvironment (ECM); this involves expression and activity of proteolytic enzymes, such as matrix-degrading proteases (MMPs) and cathepsins to degrade ECM components, as well as interactions of tumour cells with normal astrocytes [128]. By using a cellular automaton model, a model that evolves with time according to rules based on the state of neighbouring digital cells in a lattice structure, Aubert et al. investigate how gap junctions between glioma cells (homotypic) or glioma cells and surrounding astrocytes (heterotypic) affect migration [129]. They produced two versions of their model, one focused on glioma cells growing on a collagen substrate, whilst in the other they introduced a layer of astrocytes for glioma cells to grow on. The homotypic model was made from a hexagonal lattice with a glioma spheroid at its centre which was assumed the ability to ‘eject’ an unlimited number of glioma cells. These ejected cells could only move to a connecting hexagon and that hexagon had to be empty of other cells; if this was not the case then the glioma cell did not move. For the heterotypic model, two hexagonal lattices were generated, one of astrocytes and one of glioma cells. This means that an astrocyte and a glioma cell can occupy the same hexagon but on two different planes. The main difference between the homotypic and heterotypic models is that in the homotypic model, hexagons already occupied cannot be migrated into, however in the heterotypic model, hexagons already occupied by astrocytes can be re-occupied by migrating glioma cells. By using these models authors found that inhibition of homotype gap junctions favours migration, while heterotype inhibition block it, and that heterotype gap junction inhibition dominates that of homotype inhibition. This cellular automaton system used by Aubert and co-authors also included the inhibition of gap junction proteins using carbenoxolone (CBX). In vitro studies have shown that CBX promotes migration of homotypic glioma cultures, which indicates that gap junction signalling maintains cells close to one another [130]. Oliveira et al. demonstrated that when glioma cells were cultured with astrocytes (heterotypic) or when they were implanted into organotypic slice cultures the addition of CBX now inhibited the migration of glioma cells. This information indicates that glioma cells rely on heterotypic gap junctions to increase their migration through brain tissue and that astrocytes are a requirement for the migration of glioma cells. Two main abnormalities characterize the development and progression of a cancer, the continual uncontrolled proliferation of cells (benign neoplasm), which do not respond appropriately to the signals regulating normal cell behavior, and their ability to abnormally migrate and invade tissues and organs (malignant invasive tumour), that eventually will allow them to spread throughout the body. However, mechanisms underlying progression from a benign neoplasm to a malignant invasive tumours are not completely understood. Hatzikirou et al. were able to demonstrate that the ability of glioblastoma to rapidly recur after a total main resection, is not due to a mere consequence of acquired cellular mutations, but it is a more complex system. By using a growth model, they proposed that the emergence of invasiveness of cancer cells is caused by the combinations of migration/proliferation mechanisms and the oxygen shortage, i.e., hypoxia, in the environment of a growing tumour. To test this hypothesis, they utilised a lattice-gas cellular automaton system (LGCA). First, they produced a model assuming two basic phenotypes, invasive or proliferative, with low or high rates of proliferation respectively. They followed the assumption that the rate of change in cell phenotype was dependent on the rate of mutation. Following a simulated resection, where 99.9% of the tumour was removed, the remaining glioma cells were assumed to behave as invasive cells and their growth was simulated for 180 days. The recurrent tumour was two orders of magnitude smaller than the tumour prior to resection which indicates that a phenotypic switch from invasive to proliferative is required for full tumour recurrence. Following on from this, they modelled in the rate of phenotypic change based on the rate of mutation and it was calculated that to achieve a fully recurred tumour mass in 180 days would take 0.001 phenotypic changes per cell division. Hatzikirou et al. infer that to achieve this level of phenotypic change, when only factoring in mutation rate, is unrealistic and is evidence that processes other than gene mutation are responsible for the fast recurrence of resected gliomas. To test the potential role of hypoxic conditions, authors generated an LGCA model that takes into account the number of tumour cells at any particular node and whether or not there is sufficient oxygen to support them. Oxygen concentrations were assumed to be homogenous throughout the lattice model. The outcome of the simulations was that lower oxygen concentrations favoured the emergence of the invasive phenotype, which was considered the ‘fittest’ phenotype as it was able to give rise to a higher number of daughter cells than the proliferative phenotype cells under the same hypoxic conditions. This proliferative to invasive phenotype switch is proposed to occur when the tumour mass increases and since it has less oxygen availability to sustain the growth, the tumour begins to develop necrotic regions [131]. Hatzikirou et al. used their results to hypothesise that one reason for such a fast tumour recurrence following resection could be down to a sudden increase in available oxygen for the remaining tumour cells and that this drastically increases the rate of transition from the invasive phenotype to the proliferative [132].

### 6.2. Modelling Parameters of Blood–Brain Barrier Permeability

The permeability of the blood–brain barrier (BBB) presents one of the biggest barriers in the design and testing of novel brain cancer therapies. The BBB is comprised of endothelial cells that are held together by tight junctions which is what gives the capillaries of the brain such low permeability [133]. This lack of permeability is important for maintaining the composition of interstitial fluid and excluding waste products in the blood from passing into the brain [134]. Whilst this low permeability is functional under physiological conditions, it presents a unique obstacle in the face of designing effective chemotherapeutic agents that can pass into the brain and accumulate at the required concentration. In silico models of the BBB allow researchers to screen drug compounds prior to in vitro or in vivo validation.

Wang et al., made use of molecular dynamics (MD) simulations to simulate the bilayer of human brain microvascular endothelial cells (hBMECs), which forms the outer layer of the BBB [135]. This model takes into account electrostatic interactions, hydrogen bonding, van der Waals forces and lipid parameters and uses the TIP3P water model as its solvent. They moved on to first characterise some well-known small molecules in the MD simulation including ammonia, ethanol, and carbon dioxide which covers a range of different chemical features. They chose these compounds to initially test the model as they are abundant in the blood stream and can readily cross the BBB. Caffeine and ethosuximide were also studied in the MD simulation as they have moderate and low BBB permeability respectively. This testing of compounds of different chemistries and brain penetration allowed the researchers to have a direct comparison between the in-silico model and experimental validations. Transwell assays using hBMECs was set up in vitro to measure the permeability of these compounds in an experimental set up. They found that the experimental data was well in alignment with the in-silico predictions [135], and thus this model has potential to be used to screen future drug candidates for their ability to cross the BBB.

Disruption of the BBB in order to deliver chemotherapeutic agents to the brain is a potential avenue of study when considering how best to get drugs into the brain. The tight junctions of the BBB make the process of drug delivery difficult and so Goliaei et al. presented a molecular dynamics simulation using ultrasound and nanobubbles to disrupt the integrity of the BBB [136]. To do this, they generated lipid coated vesicles and inserted pairs of claudin-15 protein into their membranes. The claudin-15 pairs interacted with those of neighbouring cells to represent tight junctions within the model. The nanobubbles were represented by an area of 30 nm diameter, lacking any water molecules and within close proximity to the simulated tight junctions. Simulations were run in the presence and absence of a nanobubble to study the effects of its presence following an ultrasonic shock wave. When the nanobubble was present, the shock wave resulted in collapse of the bubble integrity and caused ‘high velocity water particles’ to strike the tight junction connections. Nanobubble collapse led to the tight junctions being completely severed and would therefore represent a disrupted BBB. In contrast to this, simulations that took place in the absence of a nanobubble showed no evidence of tight junction severing and therefore represented an intact BBB [136]. These simulations present a potentially novel mechanism for introducing targeted damage to the BBB via the use of ultrasound and nanobubbles.

Prabhakar and Banerjee, have since gone on to demonstrate the biological potential of these nanobubbles in delivering drugs to cells in vitro. By combining nanobubbles, liposomes and the chemotherapy drug, paclitaxel (PTX) they show that bursting nanobubbles with ultrasound lead to a much higher percentage of PTX being released compared to the use of nanobubbles without ultrasound. They also demonstrated that the combined use of PTX-containing nanobubbles and ultrasound caused a much higher cytotoxicity in a variety of cell lines than those without ultrasound [137]. The use of nanobubble and ultrasound therapies is an exciting new approach to deliver drugs across the BBB in a manner that reduces the overall drug dose required and would bypass a lot of the unwanted systemic side effects that come with current chemotherapy agents. It also means that drugs that have previously shown excellent anti-cancer effects but failed to cross the BBB, could now be employed by enveloping these drugs in nanobubbles.

### 6.3. Discovering Evolutionary Patterns and Understanding the Emergence of Drug Resistance

Clonal evolution is one of the main processes that drives tumour progression. The emergence of heterogeneous clones within a tumour is caused by the genetic, epigenetic, and microenvironmental selective pressure that tumour cells experience during cancer progression. Clonal evolution is also one of the major causes of therapeutic resistance to anticancer agents. Understanding the evolutionary course of an individual patient’s cancer is likely to be essential in deciding their optimal treatment plan and diagnosis and subsequent points of progression or recurrence.

In recent years, multi-region sequencing has allowed researchers to discover the temporal sequence of some genomic changes within a tumour, however the robust identification of repeated evolution across patients remains a challenge. Recently, Caravagna and co-authors have developed a new analysis tool, named REVOLVER (Repeated EVOLution in cancER), that utilises a machine learning approach named ‘transfer learning’ to jointly analyse multi-region sequencing data [138]. This novel approach was used to highlight hidden evolutionary patterns within patient cohorts. Caravagna et al., did not apply the REVOLVER algorithm to glioma samples but were able to show its power to detect evolutionary paths in a series of primary breast cancers. They were able to classify six different groups based on evolutionary trajectories and correlate them with the outcome of survival analysis. REVOLVER was able to demonstrate which evolutionary trajectories were likely to correlate with better or worse prognoses, highlighting the potential for stratifying new patients based on likely tumour evolution.

Drug resistance and tumour recurrence can also be caused by clonal evolution induced by the selective pressure during therapy. Small populations of genetically distinct subclones present in a tumour can evolve under the pressure of drug treatment, resulting in the selection of the more ‘fit’ subclones. These subclones may persist after treatment and repopulate the tumour over time, leading to disease recurrence. Resistant subclones are likely to be generated in response to therapy in two different ways: (1) drug therapies induce genetic changes in a small number of cells, generating mutant subclones, which are preferentially augmented during subsequent treatment; (2) therapy resistant subclones generated during tumour progression, are present pre-treatment and continue to thrive despite further therapy. In some cases, such as metastatic breast cancer, leukaemia, non-small cell lung cancer as well as glioblastoma [139], it has been shown that subclones containing cellular information pertinent to tumour recurrence, occurred at the early stages of initial tumour development, rather than the accumulation of mutational burden over time. Interestingly, the Glioma Longitudinal Analysis (GLASS) consortium analysed longitudinal datasets to study the clonal dynamics of gliomas in matched patient tumours, using samples taken at initial diagnosis and at recurrence. The study found that across different tumour samples there were commonalities between them over time such as the acquisition of a hypermutated phenotype. However, at the single patient level, it appeared that treatment resulted in stochastic evolution of many of the gliomas rather than predictable evolutionary traits. These data indicate that the treatment interventions for glioma do not always result in predictable tumour evolution and that the acquisition of various phenotypes at recurrence warrants further investigation [140].

Mathematical modelling of the evolution of drug resistance is of great clinical benefit as it could identify resistant subclones within a population that have resistance against certain therapies and therefore inform different clinical decisions. Evolutionary herding is the use of drugs that select for specific phenotypes, this can present differential therapy courses depending on each patient’s tumour heterogeneity. Each drug will produce ‘fitness peaks’, where cell populations unaffected by that drug will cluster, and ‘fitness valleys’ where affected cell populations will cluster. Combination therapy that selects for overlapping fitness peaks will select for multi-drug resistant cell populations. The idea behind evolutionary herding is for one drug to select for a cell population that falls into one fitness peak that forms a fitness valley under the influence of a second drug—thus leading to the elimination of the cancer cell population [141,142].

### 6.4. Virtual Screening for New Drug Molecules and Virtual Clinical Trials

Oligodendroglioma is characterised by either *IDH1* or *IDH2* mutations combined with the loss of both 1p/19q chromosomal arms. It is a relatively slow growing tumour that mostly affects younger patients, has a longer median survival time and has a better response to alkylating agents in comparison to the other diffuse gliomas [143]. Despite the available surgical and therapeutic options, it is still an incurable malignancy. Modelling oligodendroglioma growth and therapy response may represent a useful tool in ascertaining whether new and adaptive treatment regimens will be useful clinically to improve survival and the patient’s quality of life by way of reducing toxicity and side effects. A model that uses real patient data to model the parameters of glioma growth and response to TMZ was recently proposed [144]. Data from these patients was used to generate an ‘in-silico twin’ upon which simulations of differing treatment regimens could be tested. The interval time between treatment cycles was increased by intervals of 15 days up to 6 months. These data show that whilst tumour growth was shown to be similar between different treatment regimens, there were some ‘in-silico twins’ for which the alternative regimen improved survival. A clinical trial with 2000 patients was simulated, in which half received a randomised number of treatment cycles undergone by the real patients (between 5 and 18 cycles) and the other half received the newly proposed treatment regimen. This virtual clinical trial showed that the new treatment regimen extended the median survival by 3.8 years and paves the way for a clinical trial with real patients to take place to improve the treatment and management of grade II oligodendroglioma.

The use of computational modelling for drug design can provide more precise insight into the structure and chemistry required from a new compound. This can help to eliminate time spent in the lab optimising hundreds of potential compounds. Virtual drug screens can be conducted using quantitative structure-activity relationships (QSAR) and ligand-based virtual screens (LBVS) [145].

QSAR models have been previously utilised in the design and virtual testing of new EGFR inhibitors, which could provide future treatments for EGFR-amplified glioma. The study conducted by Zhao et al. employed 2D-QSAR to first ascertain whether or not a series of potential drugs would be classed as EGFR inhibitors [146]. The 2D-QSAR model was first ‘trained’ using a combined set of inhibitors and non-inhibitors and when tested against an independent data set, was found to predict EGFR inhibitors with 97.67% accuracy. Zhao et al. then went on to develop a 3D-QSAR model which measured the activity of a range of EGFR inhibitors. This allowed inhibitors with the best activities to be used in the redesign of new drugs, by combining the best molecular aspects of each compound. The combination of both 2D and 3D-QSAR models meant that new drugs could be virtually screened for their suitability as an inhibitor and also ascertain their level of activity which would be useful in narrowing down which drugs are of the highest likely benefit going forward into animal models and clinical trials. LBVS is a method that uses molecular descriptors—features of drugs known to have a certain activity—that are applied to virtual screens as filters to narrow down which drugs have the features associated with compounds already in clinical use, and therefore have a higher likelihood of making it to the clinic themselves [147]. A closely related technique is structure-based drug design that relies on known structural elements of the target to design drugs that was be best suited to interacting with it [148]. Virtual drug screening has already been used in the design of new TMZ derivatives that could offer a higher biological activity whilst reducing side effects from interactions with non-tumour tissues. The in-silico study found that out of the 10 modified TMZ-derivatives, 6 of them had more favourable absorption, distribution, metabolism and excretion (ADME) scores than TMZ itself. These 6 derivatives were tested in vitro on U87MG and U251 cell lines where three of the drugs caused a greater decrease in proliferation than TMZ alone [149]. This study indicates how computational design of drug molecules can provide new drugs that have more favourable in vitro effects.

## 7. Epigenetic Alterations Found within Glioma, from Identification of Novel Biomarkers to Predictors of Clinical Outcomes and Treatment Options

DNA methylation occurs as a covalent transfer of a methyl group onto one of the DNA nucleotide bases, either as a targeted modification via DNA methyltransferases or as an effect of DNA damaging agents [150]. Methylation at the carbon-5 position of cytosine (5-methylcytosine, 5mC) is a well-documented epigenetic DNA alteration that controls the expression of a wide range of genes as well as playing a role in chromatin structure [151,152]. The oxidation of 5mC by the TET family of dioxygenases produces 5-hydroxymethylcytosine (5hmC); this is the first step in the demethylation of DNA. The process of demethylation continues on to form the derivatives, 5-formylcytosine (5fC), and then 5-carboxylcytosine (5caC). 5caC is targeted by the base excision repair system and is replaced by an unmodified cytosine base thus arriving back at an unmethylated cytosine base [153]. 5fC and 5caC appear to have proteins that preferentially bind to them over 5mC or 5hmC, which indicates that these 5mC derivatives may have their own epigenetic signalling cascades and functions. Some transcriptional and chromatin regulators bind to 5fC when tested in murine embryonic stem cells, whilst the DNA methyltransferase DNMT1 preferentially binds to 5caC in neural progenitor cells [154]. This indicates that whilst 5mC derivatives 5fC and 5caC have a role in demethylation of DNA, they may also have epigenetic signalling functions.

Gliomas exhibit several oncogenic pathways involved in tumour onset, progression and therapeutic resistance, and like other tumours their initiation and progression is associated with epigenetic abnormalities [155]. Hence targeting the epigenome in gliomas is considered a convincing treatment modality.

### 7.1. Methylation Patterns Differ between IDH^WT^ and IDH^mut^ Gliomas

*IDH* mutations occurs most commonly at position R132 and the most common mutation in glioma results in a change to R132H. The result of this mutation is neomorphic enzyme activity that instead of producing α-ketoglutarate produces 2-hydroxyglutarate (2-HG) [14]. 2-HG acts as a competitive inhibitor of dioxygenase enzymes, including histone demethylases, resulting in changes of the genome methylation profile [17].

Multiple studies conducted on *IDH* mutant brain tumours have focused on understanding the pathways and mechanisms involved in IDH-related gliomagenesis, and in particular the role of the epigenetic modifications and effects on IDH mutant gliomas. A review by Park and Turcan [155], discusses alterations in IDH mutational status, resulting in histone modifications and remodelling of chromatin. Here, we discuss the methylation shifts between primary and recurrent glioma in the context of G-CIMP status, a phenomenon associated with IDH mutations.

Mapping of CpG sites across multiple cancer types including glioma, melanoma, cholangiocarcinoma, and acute myeloid leukaemia revealed that IDH^mut^ cancers all showed hypermethylation when compared to their IDH^WT^ counterparts. IDH^mut^ glioma also demonstrate the highest amount of genome hypermethylation in comparison to the other cancer types tested, with 19% of CpG sites methylated compared to 2–3% for the other cancers [156]. These tumours also showed the highest level of hypomethylation when compared to other cancers, indicating that IDH mutations cannot be associated with a pre-defined methylation pattern across all cancers, but instead is tissue type dependent. They provide evidence that glioma has the largest differential gene expression between IDH^WT^ and IDH^mut^ gliomas (~4200 genes) in comparison to the other cancers tested (~135–420 genes). IDH^mut^ gliomas were also enriched for sets of genes involved in the tissue development pathway as well as the immune response, which may explain why IDH^mut^ gliomas are often poorly differentiated. The effect of IDH mutations in glioma methylation changes may be influenced by the differentiation state of the cell that the mutation occurs within. Introducing IDH1^R132H^ into both differentiated human astrocytes and neural progenitor cells showed that undifferentiated NPCs were more susceptible to hypermethylation than differentiated astrocytes [156]. Although different cancers may contain the same IDH mutation, this may not result in the same phenotypic effect. Future treatment strategies should focus on the mutation in the context of a specific disease, as a successful drug targeting IDH^mut^ may not produce reproducible gains across all IDH^mut^ cancer types.

### 7.2. Methylation Shifts in Recurrent Glioma Represents a Switch to a More Aggressive Phenotype

IDH mutations in glioma produces a unique methylation pattern termed Glioma CpG Island Methylator Phenotype (G-CIMP) [157]. LGGs can be classified as either G-CIMP low or G-CIMP high, indicating a more and less aggressive tumour, respectively [158]. De Souza et al. provide evidence of changes in methylation patterns between the primary tumour and the tumour at recurrence. Patients with G-CIMP high at both presentation and tumour recurrence have a better clinical outcome than patients whose primary tumour recurs having undergone methylation changes towards G-CIMP low. In fact, patients that presented with a G-CIMP high primary tumour but progressed to a G-CIMP low tumour at recurrence, showed higher levels of ‘stemness’ in their recurrent tumours, indicating a more aggressive malignancy. The stemness index of G-CIMP low recurrent tumours strongly resembled that of the highly aggressive IDH^WT^ primary GBMs. De Souza et al. proposed that a small population of cells within IDH^mut^ primary gliomas may already have a G-CIMP low phenotype and thus contribute to the tumour’s recurrence as the more aggressive and therapy resistant phenotype [158].

The methylation shift data presented [158] indicates that large methylome changes can occur between the primary tumour and later recurrences. To understand whether any predictive biomarkers could be identified, the DNA methylation of G-CIMP high primary tumours that progressed to G-CIMP low recurrences was compared to G-CIMP high primaries that retained this methylation status at recurrence. Seven potential biomarkers were identified that represented hypomethylated CpG sites in G-CIMP high primary tumours that shifted towards hypermethylation in the G-CIMP low at tumour recurrence. Further studies into these biomarkers may improve prediction on how tumours may progress clinically, with subsequent optimisation of care, e.g., time points for imaging surveillance, commencement of oncological treatment, and provide a more selected subgroup of patients to recruit into a clinical trial.

### 7.3. Identification of a New DNA Modification in Glioblastoma

Whilst the methylation of cytosine bases is most commonly associated with epigenetics, another form of DNA modification exists, the methylation of adenine bases at the secondary amine (N^6^-mA). The N^6^-mA modification has been demonstrated in bacteria and mammals but was only first identified in the human genome very recently [159]. By using a dot blot assay and an N^6^-mA specific antibody the existence of the modification in primary human glioblastoma as well as in validated patient-derived glioblastoma cancer stem cell models was proven [160]. Levels of N6-mA were higher in human glioblastoma samples than in normal healthy tissue indicating that it may have a tumourigenic role. ALKBH1, a demethylase enzyme, was identified as a regulator of N^6^-mA modification and when this enzyme is inhibited, tumour formation decreases, along with the self-renewing capacity of the glioma stem cells. Knockdown of ALKBH1 led to a downregulation of genes involved in the hypoxia response indicating that targeting of ALKBH1 could have clinical benefit. Like the N6-mA modification itself, ALKBH1 was highly expressed in human glioblastoma when compared to the healthy brain tissue indicating that therapeutic targeting of this enzyme could more specifically target the glioma cells over the healthy cells. The presence of high ALKBH1 levels was correlated to higher glioma grades as well as poorer survival [160]. This data suggests that this novel DNA modification has potential as a clinical target in the treatment of glioblastoma.

## 8. Liquid Biopsies Could Revolutionise Detection and Diagnosis of Glioma

Diagnosis and monitoring of glioma progression involve molecular profiling of a patient’s tumour by using a sample biopsy and/or by the usage of neuroimaging techniques, such as magnetic resonance imaging (MRI) or computer tomography (CT) scans. All these techniques however have their limitations; overall the diagnostic process requires time, a lot of expensive equipment and stress and in case of tissue biopsies, it could also confer a risk of brain swelling and haemorrhages to the patient. Liquid biopsies in gliomas are a minimally invasive technique, which have great potential in all aspects of glioma management. From detection to monitoring tumour response to therapies and early recurrence. Potential biomarkers may be identified to measure disease progression. Importantly, liquid biopsies can be obtained regularly over time and can provide up to date information about the molecular heterogeneity, and longitudinal genomic evolution of the tumour from diagnosis through progression/recurrence.

Many different biological materials can be sampled from liquid biopsy of human body fluids including: circulating tumour cells (CTCs); circulating cell-free DNA (cfDNA) which in cancer patients contains circulating tumourDNA (ctDNA); circulating cell-free tumour RNA (ctRNA) containing mRNAs and mainly small RNAs; extracellular vesicles (EVs); proteins, metabolites, and tumour-educated platelets (TEPs; for a review see [161]). In glioma patients, the most widely used liquid biopsies components are derived mainly from blood and cerebrospinal fluid (CSF). In particular cell-free nucleic acids (cfDNA and cfRNA), are the most prevalent in the blood of cancer patients. Increased amount of ctDNA has been found in blood samples from cancer patients, including glioblastoma, compared to controls or to individuals with benign or low-grade tumours, and the ctDNA level has been found to positively correlate with tumour stage [161,162,163].

Recently Noushmehr H. et al., provide evidence of how a simple blood biopsy, capable of detecting cell-free DNA (cfDNA), is important for patient stratification [164]. They obtained serum samples from patients undergoing surgery for either glioma or a non-oncogenic brain disorder such as epilepsy and once normalised for genomic size, patients with brain tumours had significantly higher cfDNA relative to epilepsy patients. They also found a trend towards IDH^WT^ patients having more cfDNA than IDH^mut^, which they explained as being down to the more aggressive nature of IDH^WT^ tumours and their associated increase in BBB breakdown. Noushmehr H. et al., found that there was a genome wide change in methylation status of glioma patients compared to healthy tissues. This led them to test whether other CpG methylation sites could be detected in cfDNA. They found that not only was there a detectable serum methylome profile but that it was distinct from that of other neoplasms. The analysis of cfDNA methylome also showed that it was possible to decipher the IDH status of the tumour from the serum methylome. A simple blood test capable of identifying a glioma over other neoplasms and also being able to identify the IDH status may provide a non-intrusive way by which patients can be stratified early on in the treatment process without having to undergo invasive biopsies or time-consuming scans.

Recently, increasing interest in developing tests from cerebrospinal fluid (CSF) has been developed, since this fluid is produced in the choroid plexus of the brain and would have direct access to any waste products such as cfDNA [161]. The use of CSF could provide insight into clinically relevant biomarkers that may provide an alternate method of obtaining information with less invasive technique than the current diagnostic path of brain surgery. Whilst access to CSF may provide a higher concentration of biomarkers, it is less easy to access than a blood sample. CSF sampling requires a lumbar puncture which is more intrusive than a blood test [165]. Urine tests have been suggested for the diagnosis and tracking of glioblastoma by measuring levels of VEGF among other markers; however, these fail to be sensitive or specific enough to be of clinical use in glioblastoma [161].

## 9. Current Treatments and Future Therapy

### 9.1. Surgery for Diffuse Gliomas

Glioma surgery has been revolutionised over the last decade, due to the combination of significant advances in brain tumour imaging, intraoperative technologies and neurosurgical techniques. The philosophy of treatment, that safe, maximal glioma resection improves symptom management, quality of life, and both progression free and overall survival has also become less nihilistic. Currently, the relationship between the extent of resection (EOR) of both LGG and HGG, and clinical outcomes remain incompletely understood. There is no validated metric to quantify EOR and randomized clinical trials are impractical, which hinders achievement of level-one evidence for EOR. However, a growing body of clinical data supports the prognostic importance of gross total resection in LGG and HGG [166], and is incorporated into European guidelines [167,168]. The pursuit of maximal EOR in glioma surgery requires great caution and must be balanced against functional outcome. Failure to identify and preserve eloquent brain regions can significantly compromise the patient’s quality of life, performance status and potentially render them ineligible for further adjunctive treatment options with consequent serious prognostic implications [169,170].

Numerous intraoperative tools have been developed and evaluated, aiming to enhance the neurosurgeon’s ability to identify tumour boundaries and augment resection, while simultaneously preserving eloquent brain function.

Fluorescence guided surgery allows real-time intraoperative identification of residual tumour. The best studied intraoperative fluorescence imaging technology is 5-aminolevulinic acid (5-ALA), which is used in HGG [171]. Oral administration of the prodrug 5-ALA between 2–4 h prior to surgery results in preferential accumulation of fluorescent protoporphyrin IX (PpIX) in proliferating HGG tumour cells. PpIX is seen as bright pink under a specialised microscope with violet blue excitation light to visualize fluorescence and can guide resection. A phase three multicentre, randomized, controlled trial 5-ALA-guided resection resulted in a 29% reduction in the proportion of patients with HGG residual disease on early postoperative MRI and correlated with an increase in PFS at 6 months [171] and increased OS [172].

Cortical functional organization varies considerably between patients, tumour mass effect may distort anatomic relationships and cortical plasticity may result in reorganization of neural networks. Intraoperative neurophysiological/electrical stimulation mapping and monitoring either during awake craniotomy or under general anaesthesia, aims to reliably and reproducibly identify and localize the cortical areas and subcortical pathways involved in language, motor, sensory, and cognitive function and preserve these functions during tumour resection (for reviews of the techniques see [166,173]). Meta-analysis demonstrated that intraoperative stimulation techniques reduce late severe neurological deficits without compromise of EOR in glioma surgery when tumours arise in eloquent brain regions [174]. Schucht and colleagues demonstrated that for GBM surgery, 5-ALA guidance combined with intraoperative neurophysiological mapping and monitoring resulted in increased EOR and reduced mortality [175]. Furthermore, in cases where GBM is adjacent to the motor eloquent areas, a synergistic benefit of using intraoperative continuous dynamic subcortical mapping to identify the corticospinal tract and 5-ALA guided surgery has been demonstrated to achieve high rates of complete resection of contrast enhancing tumour [176].

Recent advances in intra-operative neurosurgical imaging including intraoperative neuronavigation, intraoperative MRI (iMRI), and intraoperative ultrasound (iUS) have significantly enhanced the potential to achieve maximal and even supra-maximal glioma resection [177,178]. Integrated multimodal neuronavigation refers to novel techniques to co-register multiple imaging modalities, including functional and structural information allowing real-time integrated intraoperative information to assist safe and complete resection of intracranial lesions particularly within eloquent brain areas. Functional MRI, MRI-based diffusion tensor imaging tractography, and navigated transcranial magnetic stimulation enable the neurosurgeon to incorporate functional data into pre-operative planning and intraoperative navigation (for a review see [166]). A recent Cochrane review of intraoperative imaging technology to maximize extent of resection for glioma concluded that while there was evidence of benefit from iMRI and 5-ALA the quality of evidence was low and impact on OS, PFS and quality of life were unclear [179]. In the context of current evidence base, the National Institute of Clinical Excellence (NICE [167]) and the European association of neurosurgical oncology guidelines (EANO [168]), advise that 5-ALA should be offered to all patients with HGG undergoing complete resection and iMRI should be considered [168].

### 9.2. Radiotherapy and Chemotherapy for Diffuse Gliomas

Following surgery, diffuse astrocytoma and oligodendroglioma patients are stratified into low risk and high risk. It is worth noting that a definition for high-risk and low-risk low grade gliomas is highly variable [180]. Patients who are deemed low-risk low grade glioma are those who underwent complete resection, are younger than 40 years of age, are of good neurological function and are classified as an IDH-mutant [168,181]. Based on the European Organisation for Research and Treatment of Cancer (EORTC) 22,845 analysis, a watch-and-wait with regular MRI scans policy is adopted [168]. It is worth noting that aside from a retrospective comparison, which showed equal survival between immediate radiotherapy versus deferred radiotherapy [182], no prospective data exist to advise treatment. According to the EORTC 22033-26033 [183] and RTOG 9802 [184,185] phase 3 trials, high-risk low grade glioma patients are those who are older than 40 years of age, had a large tumour (>5 cm) and reduced post-operative function or did not have complete resection. It is now widely accepted that IDH wildtype low grade gliomas are often treated as high risk since they tend to behave similarly to high-grade grade-IV glioblastomas [11]. In the high risk group, the current care after surgery is derived from the RTOG 9802 trial [184], which includes radiotherapy between 50–54 Gray [186,187] followed by adjuvant procarbazine, lomustine, and vincristine (PCV), therapy of six cycles.

The standard of post-operative care for high risk high-grade gliomas is based on results from three phase 3 trials (20–22) and interim result from the CATNON trial. This includes radiotherapy (60 Gray) with either six cycles of adjuvant PCV or concurrent plus adjuvant temozolamide (TMZ). The interim analysis of CATNON trial showed that non 1p19q codeleted anaplastic astrocytomas can benefit from 12 cycles of TMZ as well. The CODEL trial is currently investigating whether TMZ can replace PCV in the treatment of 1p19q codeleted oligodendrogliomas. Further, the POLCA trial is currently investigating whether radiotherapy for 1p19q codeleted oligodendrogliomas can be replaced by PCV only. For glioblastoma, radiotherapy of 60 Gray for six weeks with concurrent daily TMZ followed by six cycles of TMZ has been the standard of care since 2005 [188]. Additionally, an interim analysis showed that adding electric field therapy to adjuvant TMZ leading to five-month survival benefit, regardless of subgroup prognostic co-factors, i.e., total resection, MGMTp status [189,190].

For glioblastoma patients older than 70 years of age the Nordic [191] and the NOA-08 [192] trials suggest that the survival benefit conferred by temozolomide is largely restricted to patients with tumours harboring a methylated MGMT promoter. MGMT promoter methylation was predictive for benefit from temozolomide treatment, whereas patients with MGMT promoter unmethylated tumours displayed a trend for inferior survival.

Current systemic delivery of chemotherapy in glioma patients does not reach a high concentration at target within the glioma or the CNS, and leads to systemic side effects such as myelosuppression. Novel drug delivery systems aimed at improved delivery both spatially and temporally within the brain are actively being developed. These including injectable nanoparticles, implantable controlled-release polymer systems, convection enhanced delivery using a catheter-based approach to an agent directly into the brain tumour or parenchyma and using positive pressure infusion and BBB disruption by pulsed ultra-sound (reviewed in [193]).

Carmustine (BCNU) loaded wafers are FDA- approved and are placed along the surface of the glioma surgical resection cavity to deliver local chemotherapy for the following days to weeks. The hypothesis was based on the fact that local delivery of a chemotherapeutic agent will enable bypassing of the blood–brain barrier. Brem et al., has showed that usage of BCNU showed improvement in median survival (31 vs. 23 weeks compared to the placebo [194]). While the usage of loaded wafers has been controversial due to raising concerns of increasing infection rates [195,196] as well as the need of a near complete resection [197], more studies emerged to show survival benefit [198]. However, this has not been reproduced over the long term.

### 9.3. Immunotherapy and Targeted Therapy

In the past, the brain has been considered an immune-privileged organ, meaning the effects or at least the full effects of the immune system are not expected to occur. This has been based on the fact that no lymphatics exist and the blood–brain-barrier (BBB) prevents influx of immune regulators and cells. Though it has been increasingly recognised that this might be not entirely true. Importantly, it is appreciated that brain tumours are actively evading and suppress the immune system, by minimising expression of major histocompatibility complex (MHC) proteins, reduced cell activation (T-cell in particular), and expressing pro-apoptotic factors. Understanding the immune evading mechanisms has been an emerging and exciting new research field. Three main methods for overcoming immunosuppression have been in the forefront of research: (i) cytokine therapy, (ii) active immunotherapy, and (iii) passive immunotherapy. Cytokine therapy has mainly been focused on activating the immune system, hoping it will fight the tumour. Active immunotherapy has primarily been focused on priming the immune response against known tumour antigens by vaccination and adoptive T-cell therapies [199]. Passive immunotherapies have been those which use conjugate antibodies able to bind to tumour expressing antigens, linked to substances that can inhibit a pathway or kill the cells [200]. Currently, there are a few ongoing phase III vaccine trials (tumour lysate -NCT0045968 and dendritic cell vaccine–NCT02546102). Recently, clinical trials investigating therapies with immune checkpoint inhibitors in patients with primary brain tumours did not show improved overall survival. In the phase 3 study CheckMate 143 (NCT02017717), the effectiveness and safety of Nivolumab, a human monoclonal antibody binding to the programmed death-1 (PD-1) receptor, thereby potentiating an immune response to tumour cells, was compared with bevacizumab, the antibody against vascular endothelial growth factor (VEGF) [201]. Data obtained showed no improvement in overall survival of patients treated with nivolumab vs. bevacizumab; however, an improved survival with nivolumab (nivolumab 17.0 months vs. bevacizumab 10.1 months) was detected in patients affected by glioblastoma with methylated *MGMT* promoter and with no baseline corticosteroid use. This suggests a potential clinical benefit from a therapy with immune checkpoint inhibitors for this subgroup of patients [201]. Further immunotherapy studies in newly diagnosed patients with GBM are also ongoing in GB: Checkmate 548 (NCT02667587) is a study investigating the combined effect of TMZ and radiation therapy with nivolumab or placebo, while Checkmate 498 (NCT02617589) compares the effectiveness of nivolumab to TMZ, each given with radiation therapy.

Anti-angiogenic therapies have also failed the initial expectations to enhance survival and quality of life of GBM patients. Only one agent, the anti-VEGF antibody bevacizumab, has shown some efficacy in controlled clinical trials so far. This efficacy is, however, limited to improve progression-free survival (PFS) but not the overall survival in patients affected by primary and recurrent glioblastoma [202]. Targeting specific pathways either by extracellular receptor binding or affecting downstream targets, such as small tyrosine kinase molecules has also been an emerging therapeutic option. Oncogenic pathways involving PDGF/PDGFR and EGF/EGFR, and intracellular downstream pathways such as the PI3K/AKT/mTOR pathway have been trialled without positive results [203].

For instance recent studies designed to target the EGFR pathway in patients with *EGFR*-amplified GBMs have shown limited efficacy. The INTELLANCE-2 is a randomised controlled phase II trial investigating, the efficacy and safety of depatuxizumab mafodotin (also known as Depatux-M or as ABT-414) in patients with recurrent *EGFR*-amplified glioblastoma. Depatux-M is an antibody-drug conjugate, that targets cancer cells by linking the toxin monomethyl auristatin F (MMAF) with an antibody directed against the epidermal growth factor receptor (EGFR) or mutant EGFRvIII. In this trial the efficacy of Depatux-M was studied in combination with TMZ or as a single agent, versus TMZ or lomustine alone (NCT02343406). Data obtained from the study showed a clinical benefit of Depatux-M in combination with TMZ in recurrent *EGFR*-amplified glioblastoma, suggesting a possible role for the use of Depatux-M in combination with TMZ, especially in patients relapsing well after the end of first-line adjuvant TMZ treatment [204]. However, a companion phase III trial, the INTELLANCE-1, designed to evaluate the effectiveness and safety of Depatux-M in newly diagnosed EGFR amplified glioblastoma patients, found that the addition of Depatux-M to standard chemo-irradiation with TMZ did not demonstrate any survival advantage (NCT02573324).

Since a huge percentage of low grade gliomas and secondary glioblastomas bear mutations in *IDHs*, huge efforts have been spent pursuing the identification of novel direct inhibitors of the mutant *IDH*. AGI-5198 has been reported as the first novel, synthetic, direct enzyme inhibitor of the *IDH* mutant enzyme [205]. This drug was able to block the generation of D-2-HG, which is aberrantly produced in *IDH* mutant gliomas, impairing xenograft progression in vivo [205]. As reported, the drug was able to induce the expression of a gene that is related to differentiation, leading to reduced proliferation. AG-120 (ivosidenib) and AG-881 (vorasidenib) and AG-221 (enasidenib) are the second generations selective, reversible drug inhibitors produced, which are approved by Food and Drug Administration for the treatment of acute myeloid leukaemia [206] (https://www.fda.gov/drugs/informationondrugs/approveddrugs/ucm569482.htm). AG-221 was tested in a clinical trial for gliomas and other *IDH* mutant tumours in 2014, showing inhibitory effects for these tumours. However, appropriate dosing was an issue. Also, a number of clinical trials are underway, currently evaluating the efficacy and safety profile of AG-120 and AG-881 (NCT03343197). Since the discovery that *IDH1* R132H is the most common mutation in ‘diffuse’ gliomas, more clinical trials have emerged (NCT02073994; NCT02273739; NCT02454634; NCT02454634; NCT02771301). Recently, Agios Pharmaceuticals has conducted a multicentre clinical study on recurrent LGG with *IDH* mutation using an AG-120 and AG-881 (NCT03343197). The primary outcome was to compare the D-2-HG concentrations in surgically removed tumours, which were treated versus not treated with the drug inhibitors. Clinical safety, dosage, tolerance, and pharmacokinetics will also be studied. This safety and feasibility trial will provide appropriate dosing for future studies. It is worth noting that the new inhibitors exhibit a good CSF-plasma ratio [207].

Despite the positive results, the success of *IDH*-mutant inhibitors is found to have a plethora of limitations. A study showed that despite the fact that AGI-5198 reduces neomorphic activity, it also does not alleviate the DNA and histone hypermethylation phenotype since histone methylation was found to be high [208]. Further, Sulkowski and colleagues demonstrated that AGI-5198 is preventing DNA damage in cancer cells, leading to the conclusion that this might allow for resistance to DNA damage agents like current chemo and radiotherapeutic options [209]. This has also been confirmed by another study, showing that *IDH1* mutated cells under the action of AGI-5198 gain radioprotective abilities [210]. Currently, a number of other novel molecular inhibitors are tested and can be combined with *IDH* inhibitors to overcome possible drawbacks of each.

Further, there are currently three trials under investigation for IDH1 peptide vaccines (NCT02454634; NCT02454634; NCT02771301). Initially, a spontaneous immune response to the IDH mutation has been documented [211]. The researchers used a 15 amino acid–base construct to generate an IDH1 peptide, with the R132H mutation and injected to mice. In animal models, it was reported that IDH1 mutated cells could be prevented from growing in the CNS and the vaccine preserved the normal physiological function of IDH1 wildtype gene [211]. To date, the German National Cancer Centre, Duke University, and the Tiantan Hospital in China initiated three randomized control trials for IDH1 vaccines.

### 9.4. Future Trials and Therapies

The ability to deliver precision/stratified medicine represents a challenge in glioma patients. Future treatments will benefit from ‘fresh tumour tissue’ resected at surgery that is surplus to diagnostic requirement. The tissue must be suitable for whole genome analysis, epigenomics, transcriptomics, methylomics, and proteomics to allow identification of specific tumour targets, drug sensitivities, and immune modulatory factors. The British feasibility study of molecular stratification and targeted therapy to optimize the clinical management of patients with glioma by enhancing clinical outcomes, reducing avoidable toxicity, improving management of post-operative residual and recurrent disease and improving survivorship (The Tessa Jowell BRAIN MATRIX trial www.birmingham.ac.uk/brainmatrix) is a national platform study striving to optimise this strategy. The aim of BRAIN MATRIX is to test the hypothesis that comprehensive whole-genome sequencing and epigenomic profiling of gliomas is feasible in a timely manner in the UK, and that the results improve the stratification of patients for next generation (targeted) therapies, to ultimately improve clinical outcomes and quality of life. Furthermore, optimised fresh tumour sampling pipelines can be used to provide good quality tissue in order to create autologous vaccines and potential immunotherapies. Advanced trial techniques, such as adaptive designs, can make clinical trials more flexible by using results accumulating in the trial to modify the trial’s course in accordance with pre-specified rules. These techniques can shorten trials duration, obtain more precise conclusions however are more complex and have had success in other cancers notably lung cancer (https://clinicaltrials.gov/ct2/show/NCT02664935).

## 10. Conclusions

Future advancements in glioma treatments require a more integrated approach and close collaboration between patients, clinicians, scientists, and clinical trial units in order to incorporate translational research focusing on precision medicine and adaptive clinical trials. The use of single cell RNA-seq methods and a wider range of in vitro and in vivo models will allow the molecular events propagating glioma to be further elucidated. The role of the tumour microenvironment as well as that of normal brain cells requires further attention in order to understand how these brain-specific components influence the development, progression, and recurrence of glioma so that more comprehensive therapies can be developed. Aiding this, in silico drug design and testing allows us to quickly understand if a certain drug will pass the blood–brain barrier, a major hurdle in the development of systemically administered drugs, and whether a new drug will provide any therapeutic benefit. Our understanding of the molecular events during glioma onset and progression have greatly improved in recent years, sadly the clinical aspects of patient stratification care have not matched this. Greater co-operation between clinicians, scientists and clinical trials will, in the future, lead to more therapeutic options to combat this invariably lethal disease.

## Figures and Tables

**Table 1:** In the 2016 WHO classification of “diffuse gliomas” (grades II-IV), are classified in two groups based on the Isocitrate Dehydrogenase genes (IDH1 and 2), wildtype versus mutant status.

Cell Type	Classification	Histological Grading	Molecular Classification
‘Diffuse’ astrocytomas	Diffuse astrocytoma (A)	II	IDH wildtype (30%) IDH mutant (70%)
	Anaplastic astrocytoma (AA)	III	
	Glioblastoma (GB)	IV	IDH wildtype (90%) IDH mutant (10%)
Oligodendrogliomas	Oligodendroglioma (O)	II	IDH mutant (100%) 1p19q codeleted (100%)
	Anaplastic oligodendroglioma (AO)	III	

**Table 2:** Types of in vitro, ex vivo and in vivo models to study brain cancer (images created with BioRender.com).

	Summary	Advantages	Disadvantages
Monolayer cell culture 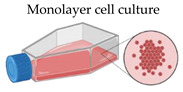	2D culture system Can be either primary or immortalised cell lines	Readily available resource Minimal cost Easy to use Allow rapid studies of biological properties of tumours Allow screening of multiple drug compounds	Genetically homogeneous Genetic and epigenetic drift from the original tumour caused by the standard serum-based in vitro conditions Impossibility to test tumour-host interactions
Neuro/tumoursphere culture 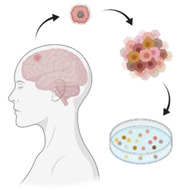	3D culture system Generated from 2D cell lines or freshly resected tumour	Preserve the complex individual level of genetic and epigenetic heterogeneity of the original tumour sample Maintain genetic stability over time Allow screening of multiple drug compounds	Represent just the cancer stem cell population of the original tumour 3D nature means that media nutrients are not equally distributed Generation depends on tumour grade and genetics Lack a realistic brain microenvironment composed by vessels and immune cells
Cerebral organoids 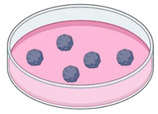	Originate from hPSCs and/or hESC Form self-organising organ structures	Recapitulates the variety of cerebral cell types Retain anatomical features of a simplified mammalian brain Allow studies of biological properties of tumours such as invasion, in a more physiological system compared to monolayer cultures	Lack a realistic brain microenvironment composed by vessels and immune cells
Organotypic brain slice culture 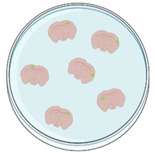	Maintain mouse brain slices in culture The genetic alteration can be introduced in the germline (knock-out, knock-in, transgenic models) or in the somatic cells (via viral-mediated gene delivery)	Maintains the in vivo brain architecture Allow faster studies of invasion ex vivo Drug screens can take place in a more realistic brain microenvironment	Not suitable for long term studies
Genetically engineered mouse models 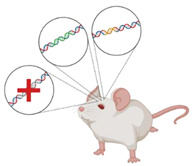	The genetic alteration can be introduced in the germline (knock-out, knock-in, transgenic models) or in the somatic cells (via viral-mediated gene delivery)	Genetic changes can be targeted to specific cell populations and induced at specific developmental stage of the animal Animals have an intact immune system Roles of individual mutations can be ascertained Tumour formation can be monitored from the very start	Only a select few genes can be altered and so does not reflect the true genetic heterogeneity Expensive and time consuming
Xenograft mouse models 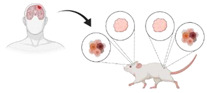	Injection of individual tumour cells or *en bloc* biopsies Tumour sample can be injected heterotopically or orthotopically	Closely resembles the original features of the tumour Heterogeneity is maintained Tumours generate their own vasculature	Animals are immune compromised so tumours do not experience input from the immune system
“Humanised” xenograft mouse models 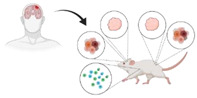	Addition of human immune cells to build a “human immune system”	Tumours experience a human immune response	Very expensive and time consuming Possibility of mice developing GVHD

## Data Availability

Data sharing not applicable. No new data were created or analyzed in this study. Data sharing is not applicable to this article.

## References

[1] Jessen K.R., Mirsky R. (1980). Glial cells in the enteric nervous system contain glial fibrillary acidic protein. Nature.

[2] Ostrom Q.T., Gittleman H., Liao P., Vecchione-Koval T., Wolinsky Y., Kruchko C., Barnholtz-Sloan J.S. (2017). CBTRUS Statistical Report: Primary brain and other central nervous system tumors diagnosed in the United States in 2010–2014. Neuro-Oncology.

[3] Lin Z., Yang R., Li K., Yi G., Li Z., Guo J., Zhang Z., Junxiang P., Liu Y., Qi S. (2020). Establishment of age group classification for risk stratification in glioma patients. BMC Neurol..

[4] Ostrom Q.T., Cioffi G., Gittleman H., Patil N., Waite K., Kruchko C., Barnholtz-Sloan J.S. (2019). CBTRUS Statistical Report: Primary Brain and Other Central Nervous System Tumors Diagnosed in the United States in 2012-2016. Neuro-Oncology.

[5] Philips A., Henshaw D.L., Lamburn G., O’Carroll M.J. (2018). Brain Tumours: Rise in Glioblastoma Multiforme Incidence in England 1995-2015 Suggests an Adverse Environmental or Lifestyle Factor. J. Environ. Public Health.

[6] Johnson D.R. (2012). Rising incidence of glioblastoma and meningioma in the United States: Projections through 2050. J. Clin. Oncol..

[7] Grech N., Dalli T., Mizzi S., Meilak L., Calleja N., Zrinzo A. (2020). Rising Incidence of Glioblastoma Multiforme in a Well-Defined Population. Cureus.

[8] Koshy M., Villano J.L., Dolecek T.A., Howard A., Mahmood U., Chmura S.J., Weichselbaum R.R., McCarthy B.J. (2012). Improved survival time trends for glioblastoma using the SEER 17 population-based registries. J. Neuro-Oncology.

[9] Claus E.B., Walsh K.M., Wiencke J.K., Molinaro A.M., Wiemels J.L., Schildkraut J.M., Bondy M.L., Berger M., Jenkins R., Wrensch M. (2015). Survival and low-grade glioma: The emergence of genetic information. Neurosurg. Focus.

[10] Louis D.N., Ohgaki H., Wiestler O.D., Cavenee W.K., Burger P.C., Jouvet A., Scheithauer B.W., Kleihues P. (2007). The 2007 WHO classification of tumours of the central nervous system. Acta Neuropathol..

[11] Louis D.N., Perry A., Reifenberger G., von Deimling A., Figarella-Branger D., Cavenee W.K., Ohgaki H., Wiestler O.D., Kleihues P., Ellison D.W. (2016). The 2016 World Health Organization Classification of Tumors of the Central Nervous System: A summary. Acta Neuropathol..

[12] Parsons D.W., Jones S., Zhang X., Lin J.C., Leary R.J., Angenendt P., Mankoo P., Carter H., Siu I.M., Gallia G.L. (2008). An integrated genomic analysis of human glioblastoma multiforme. Science.

[13] Suzuki H., Aoki K., Chiba K., Sato Y., Shiozawa Y., Shiraishi Y., Shimamura T., Niida A., Motomura K., Ohka F. (2015). Mutational landscape and clonal architecture in grade II and III gliomas. Nat. Genet..

[14] Dang L., White D.W., Gross S., Bennett B.D., Bittinger M.A., Driggers E.M., Fantin V.R., Jang H.G., Jin S., Keenan M.C. (2009). Cancer-associated IDH1 mutations produce 2-hydroxyglutarate. Nature.

[15] Lu C., Ward P.S., Kapoor G.S., Rohle D., Turcan S., Abdel-Wahab O., Edwards C.R., Khanin R., Figueroa M.E., Melnick A. (2012). IDH mutation impairs histone demethylation and results in a block to cell differentiation. Nature.

[16] Turcan S., Rohle D., Goenka A., Walsh L.A., Fang F., Yilmaz E., Campos C., Fabius A.W., Lu C., Ward P.S. (2012). IDH1 mutation is sufficient to establish the glioma hypermethylator phenotype. Nature.

[17] Xu W., Yang H., Liu Y., Yang Y., Wang P., Kim S.H., Ito S., Yang C., Wang P., Xiao M.T. (2011). Oncometabolite 2-hydroxyglutarate is a competitive inhibitor of alpha-ketoglutarate-dependent dioxygenases. Cancer Cell.

[18] Chan A.K., Yao Y., Zhang Z., Chung N.Y., Liu J.S., Li K.K., Shi Z., Chan D.T., Poon W.S., Zhou L. (2015). TERT promoter mutations contribute to subset prognostication of lower-grade gliomas. Mod. Pathol.

[19] van den Bent M.J., Brandes A.A., Taphoorn M.J., Kros J.M., Kouwenhoven M.C., Delattre J.Y., Bernsen H.J., Frenay M., Tijssen C.C., Grisold W. (2013). Adjuvant procarbazine, lomustine, and vincristine chemotherapy in newly diagnosed anaplastic oligodendroglioma: Long-term follow-up of EORTC brain tumor group study 26951. J. Clin. Oncol. Off. J. Am. Soc. Clin. Oncol..

[20] Nonoguchi N., Ohta T., Oh J.E., Kim Y.H., Kleihues P., Ohgaki H. (2013). TERT promoter mutations in primary and secondary glioblastomas. Acta Neuropathol..

[21] Simon M., Hosen I., Gousias K., Rachakonda S., Heidenreich B., Gessi M., Schramm J., Hemminki K., Waha A., Kumar R. (2015). TERT promoter mutations: A novel independent prognostic factor in primary glioblastomas. Neuro-Oncology.

[22] Hanahan D., Weinberg R.A. (2011). Hallmarks of cancer: The next generation. Cell.

[23] (2008). The Cancer Genome Atlas Research Network, Comprehensive genomic characterization defines human glioblastoma genes and core pathways. Nature.

[24] Wick W., Hartmann C., Engel C., Stoffels M., Felsberg J., Stockhammer F., Sabel M.C., Koeppen S., Ketter R., Meyermann R. (2009). NOA-04 randomized phase III trial of sequential radiochemotherapy of anaplastic glioma with procarbazine, lomustine, and vincristine or temozolomide. J. Clin. Oncol. Off. J. Am. Soc. Clin. Oncol..

[25] Weller M., Stupp R., Hegi M.E., van den Bent M., Tonn J.C., Sanson M., Wick W., Reifenberger G. (2012). Personalized care in neuro-oncology coming of age: Why we need MGMT and 1p/19q testing for malignant glioma patients in clinical practice. Neuro-Oncology.

[26] Jenkins R.B., Blair H., Ballman K.V., Giannini C., Arusell R.M., Law M., Flynn H., Passe S., Felten S., Brown P.D. (2006). A t(1;19)(q10;p10) mediates the combined deletions of 1p and 19q and predicts a better prognosis of patients with oligodendroglioma. Cancer Res..

[27] Killela P.J., Reitman Z.J., Jiao Y., Bettegowda C., Agrawal N., Diaz L.A., Friedman A.H., Friedman H., Gallia G.L., Giovanella B.C. (2013). TERT promoter mutations occur frequently in gliomas and a subset of tumors derived from cells with low rates of self-renewal. Proc. Natl. Acad. Sci. USA.

[28] Huang F.W., Hodis E., Xu M.J., Kryukov G.V., Chin L., Garraway L.A. (2013). Highly recurrent TERT promoter mutations in human melanoma. Science.

[29] Gleize V., Alentorn A., Connen de Kerillis L., Labussiere M., Nadaradjane A.A., Mundwiller E., Ottolenghi C., Mangesius S., Rahimian A., Ducray F. (2015). CIC inactivating mutations identify aggressive subset of 1p19q codeleted gliomas. Ann. Neurol..

[30] Wesseling P., van den Bent M., Perry A. (2015). Oligodendroglioma: Pathology, molecular mechanisms and markers. Acta Neuropathol..

[31] Burnet N.G., Jefferies S.J., Benson R.J., Hunt D.P., Treasure F.P. (2005). Years of life lost (YLL) from cancer is an important measure of population burden--and should be considered when allocating research funds. Br. J. Cancer.

[32] Nandakumar P., Mansouri A., Das S. (2017). The Role of ATRX in Glioma Biology. Front. Oncol..

[33] Brat D.J., Verhaak R.G., Aldape K.D., Yung W.K., Salama S.R., Cooper L.A., Rheinbay E., Miller C.R., Vitucci M., Morozova O. (2015). Comprehensive, Integrative Genomic Analysis of Diffuse Lower-Grade Gliomas. N. Engl. J. Med..

[34] Sahm F., Reuss D., Koelsche C., Capper D., Schittenhelm J., Heim S., Jones D.T., Pfister S.M., Herold-Mende C., Wick W. (2014). Farewell to oligoastrocytoma: In situ molecular genetics favor classification as either oligodendroglioma or astrocytoma. Acta Neuropathol..

[35] Wiestler B., Capper D., Sill M., Jones D.T., Hovestadt V., Sturm D., Koelsche C., Bertoni A., Schweizer L., Korshunov A. (2014). Integrated DNA methylation and copy-number profiling identify three clinically and biologically relevant groups of anaplastic glioma. Acta Neuropathol..

[36] Huse J.T., Diamond E.L., Wang L., Rosenblum M.K. (2015). Mixed glioma with molecular features of composite oligodendroglioma and astrocytoma: A true “oligoastrocytoma”?. Acta Neuropathol..

[37] Wilcox P., Li C.C., Lee M., Shivalingam B., Brennan J., Suter C.M., Kaufman K., Lum T., Buckland M.E. (2015). Oligoastrocytomas: Throwing the baby out with the bathwater?. Acta Neuropathol..

[38] Lenting K., Verhaak R., Ter Laan M., Wesseling P., Leenders W. (2017). Glioma: Experimental models and reality. Acta Neuropathol..

[39] Li N., Zhang P., Kiang K.M.Y., Cheng Y.S., Leung G.K.K. (2018). Caffeine Sensitizes U87-MG Human Glioblastoma Cells to Temozolomide through Mitotic Catastrophe by Impeding G2 Arrest. BioMed Res. Int..

[40] Schrader J., Deuster O., Rinn B., Schulz M., Kautz A., Dodel R., Meyer B., Al-Abed Y., Balakrishnan K., Reese J.P. (2009). Restoration of contact inhibition in human glioblastoma cell lines after MIF knockdown. BMC Cancer.

[41] Ernst A., Hofmann S., Ahmadi R., Becker N., Korshunov A., Engel F., Hartmann C., Felsberg J., Sabel M., Peterziel H. (2009). Genomic and expression profiling of glioblastoma stem cell-like spheroid cultures identifies novel tumor-relevant genes associated with survival. Clin. Cancer Res..

[42] Allen M., Bjerke M., Edlund H., Nelander S., Westermark B. (2016). Origin of the U87MG glioma cell line: Good news and bad news. Sci. Transl. Med..

[43] Huszthy P.C., Daphu I., Niclou S.P., Stieber D., Nigro J.M., Sakariassen P.O., Miletic H., Thorsen F., Bjerkvig R. (2012). In vivo models of primary brain tumors: Pitfalls and perspectives. Neuro-Oncology.

[44] Torsvik A., Stieber D., Enger P.O., Golebiewska A., Molven A., Svendsen A., Westermark B., Niclou S.P., Olsen T.K., Chekenya Enger M. (2014). U-251 revisited: Genetic drift and phenotypic consequences of long-term cultures of glioblastoma cells. Cancer Med..

[45] Reynolds B.A., Weiss S. (1992). Generation of neurons and astrocytes from isolated cells of the adult mammalian central nervous system. Science.

[46] Reynolds B.A., Tetzlaff W., Weiss S. (1992). A multipotent EGF-responsive striatal embryonic progenitor cell produces neurons and astrocytes. J. Neurosci.

[47] Vescovi A.L., Reynolds B.A., Fraser D.D., Weiss S. (1993). bFGF regulates the proliferative fate of unipotent (neuronal) and bipotent (neuronal/astroglial) EGF-generated CNS progenitor cells. Neuron.

[48] Balvers R.K., Kleijn A., Kloezeman J.J., French P.J., Kremer A., van den Bent M.J., Dirven C.M., Leenstra S., Lamfers M.L. (2013). Serum-free culture success of glial tumors is related to specific molecular profiles and expression of extracellular matrix-associated gene modules. Neuro-Oncology.

[49] Conti L., Pollard S.M., Gorba T., Reitano E., Toselli M., Biella G., Sun Y., Sanzone S., Ying Q.L., Cattaneo E. (2005). Niche-independent symmetrical self-renewal of a mammalian tissue stem cell. PLoS Biol..

[50] Brewer G.J., Torricelli J.R., Evege E.K., Price P.J. (1993). Optimized survival of hippocampal neurons in B27-supplemented Neurobasal, a new serum-free medium combination. J. Neurosci. Res..

[51] Lee J., Kotliarova S., Kotliarov Y., Li A., Su Q., Donin N.M., Pastorino S., Purow B.W., Christopher N., Zhang W. (2006). Tumor stem cells derived from glioblastomas cultured in bFGF and EGF more closely mirror the phenotype and genotype of primary tumors than do serum-cultured cell lines. Cancer Cell.

[52] Kim H., Xu R., Padmashri R., Dunaevsky A., Liu Y., Dreyfus C.F., Jiang P. (2019). Pluripotent Stem Cell-Derived Cerebral Organoids Reveal Human Oligodendrogenesis with Dorsal and Ventral Origins. Stem Cell Rep..

[53] Jin M.Z., Han R.R., Qiu G.Z., Ju X.C., Lou G., Jin W.L. (2018). Organoids: An intermediate modeling platform in precision oncology. Cancer Lett..

[54] Qian X., Song H., Ming G.L. (2019). Brain organoids: Advances, applications and challenges. Development.

[55] Linkous A., Balamatsias D., Snuderl M., Edwards L., Miyaguchi K., Milner T., Reich B., Cohen-Gould L., Storaska A., Nakayama Y. (2019). Modeling Patient-Derived Glioblastoma with Cerebral Organoids. Cell Rep..

[56] Xu H., Lyu X., Yi M., Zhao W., Song Y., Wu K. (2018). Organoid technology and applications in cancer research. J. Hematol. Oncol..

[57] Lancaster M.A., Knoblich J.A. (2014). Generation of cerebral organoids from human pluripotent stem cells. Nat. Protoc..

[58] Ogawa J., Pao G.M., Shokhirev M.N., Verma I.M. (2018). Glioblastoma Model Using Human Cerebral Organoids. Cell Rep..

[59] Krieger T.G., Tirier S.M., Park J., Eisemann T., Peterziel H., Angel P., Eils R., Conrad C. (2020). Modeling glioblastoma invasion using human brain organoids and single-cell transcriptomics. Neuro-Oncology.

[60] Matsumura H., Ohnishi T., Kanemura Y., Maruno M., Yoshimine T. (2000). Quantitative analysis of glioma cell invasion by confocal laser scanning microscopy in a novel brain slice model. Biochem. Biophys. Res. Commun..

[61] Jensen S.S., Meyer M., Petterson S.A., Halle B., Rosager A.M., Aaberg-Jessen C., Thomassen M., Burton M., Kruse T.A., Kristensen B.W. (2016). Establishment and Characterization of a Tumor Stem Cell-Based Glioblastoma Invasion Model. PLoS ONE.

[62] Minami N., Maeda Y., Shibao S., Arima Y., Ohka F., Kondo Y., Maruyama K., Kusuhara M., Sasayama T., Kohmura E. (2017). Organotypic brain explant culture as a drug evaluation system for malignant brain tumors. Cancer Med..

[63] Marques-Torrejon M.A., Gangoso E., Pollard S.M. (2018). Modelling glioblastoma tumour-host cell interactions using adult brain organotypic slice co-culture. Dis. Model. Mech..

[64] Eisemann T., Costa B., Strelau J., Mittelbronn M., Angel P., Peterziel H. (2018). An advanced glioma cell invasion assay based on organotypic brain slice cultures. BMC Cancer.

[65] Fomchenko E.I., Holland E.C. (2006). Mouse models of brain tumors and their applications in preclinical trials. Clin. Cancer Res..

[66] Kijima N., Kanemura Y., De Vleeschouwer S. (2017). Mouse Models of Glioblastoma. Glioblastoma.

[67] Simeonova I., Huillard E. (2014). In vivo models of brain tumors: Roles of genetically engineered mouse models in understanding tumor biology and use in preclinical studies. Cell Mol. Life Sci..

[68] Reilly K.M., Loisel D.A., Bronson R.T., McLaughlin M.E., Jacks T. (2000). Nf1;Trp53 mutant mice develop glioblastoma with evidence of strain-specific effects. Nat. Genet..

[69] Ding H., Roncari L., Shannon P., Wu X., Lau N., Karaskova J., Gutmann D.H., Squire J.A., Nagy A., Guha A. (2001). Astrocyte-specific expression of activated p21-ras results in malignant astrocytoma formation in a transgenic mouse model of human gliomas. Cancer Res..

[70] Marumoto T., Tashiro A., Friedmann-Morvinski D., Scadeng M., Soda Y., Gage F.H., Verma I.M. (2009). Development of a novel mouse glioma model using lentiviral vectors. Nat. Med..

[71] Ding H., Shannon P., Lau N., Wu X., Roncari L., Baldwin R.L., Takebayashi H., Nagy A., Gutmann D.H., Guha A. (2003). Oligodendrogliomas result from the expression of an activated mutant epidermal growth factor receptor in a RAS transgenic mouse astrocytoma model. Cancer Res..

[72] Ben-David U., Ha G., Tseng Y.-Y., Greenwald N.F., Oh C., Shih J., McFarland J.M., Wong B., Boehm J.S., Beroukhim R. (2017). Patient-derived xenografts undergo mouse-specific tumor evolution. Nat. Genet..

[73] Brandenburg S., Muller A., Turkowski K., Radev Y.T., Rot S., Schmidt C., Bungert A.D., Acker G., Schorr A., Hippe A. (2016). Resident microglia rather than peripheral macrophages promote vascularization in brain tumors and are source of alternative pro-angiogenic factors. Acta Neuropathol..

[74] Abels E.R., Maas S.L.N., Nieland L., Wei Z., Cheah P.S., Tai E., Kolsteeg C.J., Dusoswa S.A., Ting D.T., Hickman S. (2019). Glioblastoma-Associated Microglia Reprogramming Is Mediated by Functional Transfer of Extracellular miR-21. Cell Rep..

[75] Richmond A., Su Y. (2008). Mouse xenograft models vs GEM models for human cancer therapeutics. Dis. Model. Mech..

[76] Pierce A.M., Keating A.K. (2014). Creating anatomically accurate and reproducible intracranial xenografts of human brain tumors. J. Vis. Exp..

[77] Irtenkauf S.M., Sobiechowski S., Hasselbach L.A., Nelson K.K., Transou A.D., Carlton E.T., Mikkelsen T., deCarvalho A.C. (2017). Optimization of Glioblastoma Mouse Orthotopic Xenograft Models for Translational Research. Comp. Med..

[78] Lee J., Jo D.H., Kim J.H., Cho C.S., Han J.E., Kim Y., Park H., Yoo S.H., Yu Y.S., Moon H.E. (2019). Development of a patient-derived xenograft model of glioblastoma via intravitreal injection in mice. Exp. Mol. Med..

[79] Chen Q., Wang J., Liu W.N., Zhao Y. (2019). Cancer Immunotherapies and Humanized Mouse Drug Testing Platforms. Transl Oncol.

[80] Allen T.M., Brehm M.A., Bridges S., Ferguson S., Kumar P., Mirochnitchenko O., Palucka K., Pelanda R., Sanders-Beer B., Shultz L.D. (2019). Humanized immune system mouse models: Progress, challenges and opportunities. Nat. Immunol..

[81] Bernard D., Peakman M., Hayday A.C. (2008). Establishing humanized mice using stem cells: Maximizing the potential. Clin. Exp. Immunol..

[82] Nassereddine S., Rafei H., Elbahesh E., Tabbara I. (2017). Acute Graft Versus Host Disease: A Comprehensive Review. Anticancer Res..

[83] Zhao Y., Shuen T.W.H., Toh T.B., Chan X.Y., Liu M., Tan S.Y., Fan Y., Yang H., Lyer S.G., Bonney G.K. (2018). Development of a new patient-derived xenograft humanised mouse model to study human-specific tumour microenvironment and immunotherapy. Gut.

[84] Semenkow S., Li S., Kahlert U.D., Raabe E.H., Xu J., Arnold A., Janowski M., Oh B.C., Brandacher G., Bulte J.W.M. (2017). An immunocompetent mouse model of human glioblastoma. Oncotarget.

[85] Cao Y., Sundgren P.C., Tsien C.I., Chenevert T.T., Junck L. (2006). Physiologic and metabolic magnetic resonance imaging in gliomas. J. Clin. Oncol. Off. J. Am. Soc. Clin. Oncol..

[86] Sarkaria J.N., Hu L.S., Parney I.F., Pafundi D.H., Brinkmann D.H., Laack N.N., Giannini C., Burns T.C., Kizilbash S.H., Laramy J.K. (2018). Is the blood-brain barrier really disrupted in all glioblastomas? A critical assessment of existing clinical data. Neuro-Oncology.

[87] Greaves M., Maley C.C. (2012). Clonal evolution in cancer. Nature.

[88] Gillies R.J., Verduzco D., Gatenby R.A. (2012). Evolutionary dynamics of carcinogenesis and why targeted therapy does not work. Nat. Rev. Cancer.

[89] Marusyk A., Almendro V., Polyak K. (2012). Intra-tumour heterogeneity: A looking glass for cancer?. Nat. Rev. Cancer.

[90] Gerlinger M., Rowan A.J., Horswell S., Math M., Larkin J., Endesfelder D., Gronroos E., Martinez P., Matthews N., Stewart A. (2012). Intratumor heterogeneity and branched evolution revealed by multiregion sequencing. N. Engl. J. Med..

[91] Sottoriva A., Spiteri I., Piccirillo S.G., Touloumis A., Collins V.P., Marioni J.C., Curtis C., Watts C., Tavare S. (2013). Intratumor heterogeneity in human glioblastoma reflects cancer evolutionary dynamics. Proc. Natl. Acad. Sci. USA.

[92] Snuderl M., Fazlollahi L., Le L.P., Nitta M., Zhelyazkova B.H., Davidson C.J., Akhavanfard S., Cahill D.P., Aldape K.D., Betensky R.A. (2011). Mosaic amplification of multiple receptor tyrosine kinase genes in glioblastoma. Cancer Cell.

[93] Piccirillo S.G., Spiteri I., Sottoriva A., Touloumis A., Ber S., Price S.J., Heywood R., Francis N.J., Howarth K.D., Collins V.P. (2015). Contributions to drug resistance in glioblastoma derived from malignant cells in the sub-ependymal zone. Cancer Res..

[94] Patel A.P., Tirosh I., Trombetta J.J., Shalek A.K., Gillespie S.M., Wakimoto H., Cahill D.P., Nahed B.V., Curry W.T., Martuza R.L. (2014). Single-cell RNA-seq highlights intratumoral heterogeneity in primary glioblastoma. Science.

[95] Chen J., Li Y., Yu T.S., McKay R.M., Burns D.K., Kernie S.G., Parada L.F. (2012). A restricted cell population propagates glioblastoma growth after chemotherapy. Nature.

[96] Li Z., Bao S., Wu Q., Wang H., Eyler C., Sathornsumetee S., Shi Q., Cao Y., Lathia J., McLendon R.E. (2009). Hypoxia-inducible factors regulate tumorigenic capacity of glioma stem cells. Cancer Cell.

[97] Mandel J.J., Yust-Katz S., Patel A.J., Cachia D., Liu D., Park M., Yuan Y., Kent T.A., de Groot J.F. (2018). Inability of positive phase II clinical trials of investigational treatments to subsequently predict positive phase III clinical trials in glioblastoma. Neuro-Oncology.

[98] Mandel J.J., Youssef M., Ludmir E., Yust-Katz S., Patel A.J., De Groot J.F. (2018). Highlighting the need for reliable clinical trials in glioblastoma. Expert Rev. Anticancer.

[99] Olar A., Aldape K.D. (2014). Using the molecular classification of glioblastoma to inform personalized treatment. J. Pathol.

[100] Venteicher A.S., Tirosh I., Hebert C., Yizhak K., Neftel C., Filbin M.G., Hovestadt V., Escalante L.E., Shaw M.L., Rodman C. (2017). Decoupling genetics, lineages, and microenvironment in IDH-mutant gliomas by single-cell RNA-seq. Science.

[101] Cooks T., Harris C.C., Oren M. (2014). Caught in the cross fire: p53 in inflammation. Carcinogenesis.

[102] Verhaak R.G., Hoadley K.A., Purdom E., Wang V., Qi Y., Wilkerson M.D., Miller C.R., Ding L., Golub T., Mesirov J.P. (2010). Integrated genomic analysis identifies clinically relevant subtypes of glioblastoma characterized by abnormalities in PDGFRA, IDH1, EGFR, and NF1. Cancer Cell.

[103] Huse J.T., Phillips H.S., Brennan C.W. (2011). Molecular subclassification of diffuse gliomas: Seeing order in the chaos. Glia.

[104] Zheng S., Chheda M.G., Verhaak R.G. (2012). Studying a complex tumor: Potential and pitfalls. Cancer J..

[105] Bhat K.P.L., Balasubramaniyan V., Vaillant B., Ezhilarasan R., Hummelink K., Hollingsworth F., Wani K., Heathcock L., James J.D., Goodman L.D. (2013). Mesenchymal differentiation mediated by NF-kappaB promotes radiation resistance in glioblastoma. Cancer Cell.

[106] Ozawa T., Riester M., Cheng Y.K., Huse J.T., Squatrito M., Helmy K., Charles N., Michor F., Holland E.C. (2014). Most human non-GCIMP glioblastoma subtypes evolve from a common proneural-like precursor glioma. Cancer Cell.

[107] Wolf D.A., Hesterman J.Y., Sullivan J.M., Orcutt K.D., Silva M.D., Lobo M., Wellman T., Hoppin J., Verma A. (2016). Dynamic dual-isotope molecular imaging elucidates principles for optimizing intrathecal drug delivery. JCI Insight.

[108] Hambardzumyan D., Gutmann D.H., Kettenmann H. (2016). The role of microglia and macrophages in glioma maintenance and progression. Nat. Neurosci..

[109] Yan J., Kong L.Y., Hu J., Gabrusiewicz K., Dibra D., Xia X., Heimberger A.B., Li S. (2015). FGL2 as a Multimodality Regulator of Tumor-Mediated Immune Suppression and Therapeutic Target in Gliomas. J. Natl. Cancer Inst..

[110] Murray P.J., Allen J.E., Biswas S.K., Fisher E.A., Gilroy D.W., Goerdt S., Gordon S., Hamilton J.A., Ivashkiv L.B., Lawrence T. (2014). Macrophage activation and polarization: Nomenclature and experimental guidelines. Immunity.

[111] Komohara Y., Ohnishi K., Kuratsu J., Takeya M. (2008). Possible involvement of the M2 anti-inflammatory macrophage phenotype in growth of human gliomas. J. Pathol..

[112] Huettner C., Czub S., Kerkau S., Roggendorf W., Tonn J.C. (1997). Interleukin 10 is expressed in human gliomas in vivo and increases glioma cell proliferation and motility in vitro. Anticancer Res..

[113] Wagner S., Czub S., Greif M., Vince G.H., Suss N., Kerkau S., Rieckmann P., Roggendorf W., Roosen K., Tonn J.C. (1999). Microglial/macrophage expression of interleukin 10 in human glioblastomas. Int. J. Cancer.

[114] Doucette T., Rao G., Rao A., Shen L., Aldape K., Wei J., Dziurzynski K., Gilbert M., Heimberger A.B. (2013). Immune heterogeneity of glioblastoma subtypes: Extrapolation from the cancer genome atlas. Cancer Immunol Res..

[115] Wang Q., Hu B., Hu X., Kim H., Squatrito M., Scarpace L., deCarvalho A.C., Lyu S., Li P., Li Y. (2017). Tumor Evolution of Glioma-Intrinsic Gene Expression Subtypes Associates with Immunological Changes in the Microenvironment. Cancer Cell.

[116] Le L.Q., Shipman T., Burns D.K., Parada L.F. (2009). Cell of origin and microenvironment contribution for NF1-associated dermal neurofibromas. Cell Stem Cell.

[117] Hunter C., Smith R., Cahill D.P., Stephens P., Stevens C., Teague J., Greenman C., Edkins S., Bignell G., Davies H. (2006). A hypermutation phenotype and somatic MSH6 mutations in recurrent human malignant gliomas after alkylator chemotherapy. Cancer Res..

[118] Kim H., Zheng S., Amini S.S., Virk S.M., Mikkelsen T., Brat D.J., Grimsby J., Sougnez C., Muller F., Hu J. (2015). Whole-genome and multisector exome sequencing of primary and post-treatment glioblastoma reveals patterns of tumor evolution. Genome Res..

[119] Schumacher T.N., Schreiber R.D. (2015). Neoantigens in cancer immunotherapy. Science.

[120] Sharma P., Allison J.P. (2015). The future of immune checkpoint therapy. Science.

[121] Ruffell B., Coussens L.M. (2015). Macrophages and therapeutic resistance in cancer. Cancer Cell.

[122] Pyonteck S.M., Akkari L., Schuhmacher A.J., Bowman R.L., Sevenich L., Quail D.F., Olson O.C., Quick M.L., Huse J.T., Teijeiro V. (2013). CSF-1R inhibition alters macrophage polarization and blocks glioma progression. Nat. Med..

[123] Ries C.H., Cannarile M.A., Hoves S., Benz J., Wartha K., Runza V., Rey-Giraud F., Pradel L.P., Feuerhake F., Klaman I. (2014). Targeting tumor-associated macrophages with anti-CSF-1R antibody reveals a strategy for cancer therapy. Cancer Cell.

[124] Lun M., Lok E., Gautam S., Wu E., Wong E.T. (2011). The natural history of extracranial metastasis from glioblastoma multiforme. J. Neuro-Oncology.

[125] Cuddapah V.A., Robel S., Watkins S., Sontheimer H. (2014). A neurocentric perspective on glioma invasion. Nat. Rev. Neurosci..

[126] Noroxe D.S., Poulsen H.S., Lassen U. (2016). Hallmarks of glioblastoma: A systematic review. Esmo Open.

[127] Alfonso J.C.L., Talkenberger K., Seifert M., Klink B., Hawkins-Daarud A., Swanson K.R., Hatzikirou H., Deutsch A. (2017). The biology and mathematical modelling of glioma invasion: A review. J. R Soc. Interface.

[128] Demuth T., Berens M.E. (2004). Molecular mechanisms of glioma cell migration and invasion. J. Neuro-Oncology.

[129] Aubert M., Badoual M., Christov C., Grammaticos B. (2008). A model for glioma cell migration on collagen and astrocytes. J. R Soc. Interface.

[130] Oliveira R., Christov C., Guillamo J.S., de Bouard S., Palfi S., Venance L., Tardy M., Peschanski M. (2005). Contribution of gap junctional communication between tumor cells and astroglia to the invasion of the brain parenchyma by human glioblastomas. BMC Cell Biol..

[131] Monteiro A.R., Hill R., Pilkington G.J., Madureira P.A. (2017). The Role of Hypoxia in Glioblastoma Invasion. Cells.

[132] Hatzikirou H., Basanta D., Simon M., Schaller K., Deutsch A. (2012). ’Go or grow’: The key to the emergence of invasion in tumour progression?. Math. Med. Biol..

[133] Sweeney M.D., Sagare A.P., Zlokovic B.V. (2018). Blood-brain barrier breakdown in Alzheimer disease and other neurodegenerative disorders. Nat. Rev. Neurol..

[134] Zhao Z., Nelson A.R., Betsholtz C., Zlokovic B.V. (2015). Establishment and Dysfunction of the Blood-Brain Barrier. Cell.

[135] Wang Y., Gallagher E., Jorgensen C., Troendle E.P., Hu D., Searson P.C., Ulmschneider M.B. (2019). An experimentally validated approach to calculate the blood-brain barrier permeability of small molecules. Sci. Rep..

[136] Goliaei A., Adhikari U., Berkowitz M.L. (2015). Opening of the blood-brain barrier tight junction due to shock wave induced bubble collapse: A molecular dynamics simulation study. ACS Chem. Neurosci..

[137] Prabhakar A., Banerjee R. (2019). Nanobubble Liposome Complexes for Diagnostic Imaging and Ultrasound-Triggered Drug Delivery in Cancers: A Theranostic Approach. ACS Omega.

[138] Caravagna G., Giarratano Y., Ramazzotti D., Tomlinson I., Graham T.A., Sanguinetti G., Sottoriva A. (2018). Detecting repeated cancer evolution from multi-region tumor sequencing data. Nat. Methods.

[139] Brady S.W., McQuerry J.A., Qiao Y., Piccolo S.R., Shrestha G., Jenkins D.F., Layer R.M., Pedersen B.S., Miller R.H., Esch A. (2017). Combating subclonal evolution of resistant cancer phenotypes. Nat. Commun..

[140] Barthel F.P., Johnson K.C., Varn F.S., Moskalik A.D., Tanner G., Kocakavuk E., Anderson K.J., Abiola O., Aldape K., Alfaro K.D. (2019). Longitudinal molecular trajectories of diffuse glioma in adults. Nature.

[141] Zhao B., Hemann M.T., Lauffenburger D.A. (2016). Modeling Tumor Clonal Evolution for Drug Combinations Design. Trends Cancer.

[142] Acar A., Nichol D., Fernandez-Mateos J., Cresswell G.D., Barozzi I., Hong S.P., Spiteri I., Stubbs M., Burke R., Stewart A. (2019). Exploiting evolutionary herding to control drug resistance in cancer. BioRxiv.

[143] Grier J.T., Batchelor T. (2006). Low-grade gliomas in adults. Oncologist.

[144] Perez-Garcia V.M., Ayala-Hernandez L.E., Belmonte-Beitia J., Schucht P., Murek M., Raabe A., Sepulveda J. (2019). Computational design of improved standardized chemotherapy protocols for grade II oligodendrogliomas. PLoS Comput Biol..

[145] Miranda A., Cova T., Sousa J., Vitorino C., Pais A. (2018). Computational modeling in glioblastoma: From the prediction of blood-brain barrier permeability to the simulation of tumor behavior. Future Med. Chem..

[146] Zhao M., Wang L., Zheng L., Zhang M., Qiu C., Zhang Y., Du D., Niu B. (2017). 2D-QSAR and 3D-QSAR Analyses for EGFR Inhibitors. BioMed Res. Int..

[147] Ferreira L.G., Dos Santos R.N., Oliva G., Andricopulo A.D. (2015). Molecular docking and structure-based drug design strategies. Molecules.

[148] Acharya C., Coop A., Polli J.E., Mackerell A.D. (2011). Recent advances in ligand-based drug design: Relevance and utility of the conformationally sampled pharmacophore approach. Curr. Comput. Aided Drug Des..

[149] Yang B., Li X., He L., Zhu Y. (2018). Computer-aided design of temozolomide derivatives based on alkylglycerone phosphate synthase structure with isothiocyanate and their pharmacokinetic/toxicity prediction and anti-tumor activity in vitro. Biomed. Rep..

[150] Shen L., Song C.X., He C., Zhang Y. (2014). Mechanism and function of oxidative reversal of DNA and RNA methylation. Annu. Rev. Biochem.

[151] Breiling A., Lyko F. (2015). Epigenetic regulatory functions of DNA modifications: 5-methylcytosine and beyond. Epigenetics Chromatin.

[152] Jang H.S., Shin W.J., Lee J.E., Do J.T. (2017). CpG and Non-CpG Methylation in Epigenetic Gene Regulation and Brain Function. Genes.

[153] Kohli R.M., Zhang Y. (2013). TET enzymes, TDG and the dynamics of DNA demethylation. Nature.

[154] Iurlaro M., Ficz G., Oxley D., Raiber E.A., Bachman M., Booth M.J., Andrews S., Balasubramanian S., Reik W. (2013). A screen for hydroxymethylcytosine and formylcytosine binding proteins suggests functions in transcription and chromatin regulation. Genome Biol..

[155] Park J.W., Turcan S. (2019). Epigenetic Reprogramming for Targeting IDH-Mutant Malignant Gliomas. Cancers.

[156] Unruh D., Zewde M., Buss A., Drumm M.R., Tran A.N., Scholtens D.M., Horbinski C. (2019). Methylation and transcription patterns are distinct in IDH mutant gliomas compared to other IDH mutant cancers. Sci. Rep..

[157] Gusyatiner O., Hegi M.E. (2018). Glioma epigenetics: From subclassification to novel treatment options. Semin Cancer Biol..

[158] de Souza C.F., Sabedot T.S., Malta T.M., Stetson L., Morozova O., Sokolov A., Laird P.W., Wiznerowicz M., Iavarone A., Snyder J. (2018). A Distinct DNA Methylation Shift in a Subset of Glioma CpG Island Methylator Phenotypes during Tumor Recurrence. Cell Rep..

[159] Xiao C.L., Zhu S., He M., Chen D., Zhang Q., Chen Y., Yu G., Liu J., Xie S.Q., Luo F. (2018). N(6)-Methyladenine DNA Modification in the Human Genome. Mol. Cell.

[160] Xie Q., Wu T.P., Gimple R.C., Li Z., Prager B.C., Wu Q., Yu Y., Wang P., Wang Y., Gorkin D.U. (2018). N(6)-methyladenine DNA Modification in Glioblastoma. Cell.

[161] Saenz-Antonanzas A., Auzmendi-Iriarte J., Carrasco-Garcia E., Moreno-Cugnon L., Ruiz I., Villanua J., Egana L., Otaegui D., Sampron N., Matheu A. (2019). Liquid Biopsy in Glioblastoma: Opportunities, Applications and Challenges. Cancers.

[162] Shankar G.M., Balaj L., Stott S.L., Nahed B., Carter B.S. (2017). Liquid biopsy for brain tumors. Expert Rev. Mol. Diagn.

[163] Pantel K. (2016). Blood-Based Analysis of Circulating Cell-Free DNA and Tumor Cells for Early Cancer Detection. PLoS Med..

[164] Noushmehr H., Sabedot T., Malta T., Nelson K., Snyder J., Wells M., deCarvalho A., Mukherjee A., Chitale D., Mosella M. (2019). Detection of glioma and prognostic subtypes by non-invasive circulating cell-free DNA methylation markers. bioRxiv.

[165] Teunissen C.E., Petzold A., Bennett J.L., Berven F.S., Brundin L., Comabella M., Franciotta D., Frederiksen J.L., Fleming J.O., Furlan R. (2009). A consensus protocol for the standardization of cerebrospinal fluid collection and biobanking. Neurology.

[166] Sanai N., Berger M.S. (2018). Surgical oncology for gliomas: The state of the art. Nat. Rev. Clin. Oncol..

[167] National Guideline A. (2018). National Guideline, A. National Institute for Health and Care Excellence: Clinical Guidelines. Brain Tumours (Primary) and Brain Metastases in Adults.

[168] Weller M., van den Bent M., Tonn J.C., Stupp R., Preusser M., Cohen-Jonathan-Moyal E., Henriksson R., Le Rhun E., Balana C., Chinot O. (2017). European Association for Neuro-Oncology (EANO) guideline on the diagnosis and treatment of adult astrocytic and oligodendroglial gliomas. Lancet Oncol..

[169] Gulati S., Jakola A.S., Nerland U.S., Weber C., Solheim O. (2011). The risk of getting worse: Surgically acquired deficits, perioperative complications, and functional outcomes after primary resection of glioblastoma. World Neurosurg..

[170] Rahman M., Abbatematteo J., De Leo E.K., Kubilis P.S., Vaziri S., Bova F., Sayour E., Mitchell D., Quinones-Hinojosa A. (2017). The effects of new or worsened postoperative neurological deficits on survival of patients with glioblastoma. J. Neurosurg..

[171] Stummer W., Pichlmeier U., Meinel T., Wiestler O.D., Zanella F., Reulen H.J. (2006). Fluorescence-guided surgery with 5-aminolevulinic acid for resection of malignant glioma: A randomised controlled multicentre phase III trial. Lancet Oncol..

[172] Pichlmeier U., Bink A., Schackert G., Stummer W. (2008). Resection and survival in glioblastoma multiforme: An RTOG recursive partitioning analysis of ALA study patients. Neuro-Oncology.

[173] Szelényi A., Bello L., Duffau H., Fava E., Feigl G.C., Galanda M., Neuloh G., Signorelli F., Sala F. (2010). Intraoperative electrical stimulation in awake craniotomy: Methodological aspects of current practice. Neurosurg. Focus.

[174] De Witt Hamer P.C., Robles S.G., Zwinderman A.H., Duffau H., Berger M.S. (2012). Impact of intraoperative stimulation brain mapping on glioma surgery outcome: A meta-analysis. J. Clin. Oncol. Off. J. Am. Soc. Clin. Oncol..

[175] Schucht P., Beck J., Abu-Isa J., Andereggen L., Murek M., Seidel K., Stieglitz L., Raabe A. (2012). Gross total resection rates in contemporary glioblastoma surgery: Results of an institutional protocol combining 5-aminolevulinic acid intraoperative fluorescence imaging and brain mapping. Neurosurgery.

[176] Schucht P., Seidel K., Beck J., Murek M., Jilch A., Wiest R., Fung C., Raabe A. (2014). Intraoperative monopolar mapping during 5-ALA-guided resections of glioblastomas adjacent to motor eloquent areas: Evaluation of resection rates and neurological outcome. Neurosurg. Focus.

[177] Senft C., Bink A., Franz K., Vatter H., Gasser T., Seifert V. (2011). Intraoperative MRI guidance and extent of resection in glioma surgery: A randomised, controlled trial. Lancet Oncol..

[178] Mahboob S., McPhillips R., Qiu Z., Jiang Y., Meggs C., Schiavone G., Button T., Desmulliez M., Demore C., Cochran S. (2016). Intraoperative Ultrasound-Guided Resection of Gliomas: A Meta-Analysis and Review of the Literature. World Neurosurg..

[179] Jenkinson M.D., Barone D.G., Bryant A., Vale L., Bulbeck H., Lawrie T.A., Hart M.G., Watts C. (2018). Intraoperative imaging technology to maximise extent of resection for glioma. Cochrane Database Syst. Rev..

[180] Gorlia T., Wu W., Wang M., Baumert B.G., Mehta M., Buckner J.C., Shaw E., Brown P., Stupp R., Galanis E. (2013). New validated prognostic models and prognostic calculators in patients with low-grade gliomas diagnosed by central pathology review: A pooled analysis of EORTC/RTOG/NCCTG phase III clinical trials. Neuro-Oncology.

[181] Buckner J., Giannini C., Eckel-Passow J., Lachance D., Parney I., Laack N., Jenkins R. (2017). Management of diffuse low-grade gliomas in adults - use of molecular diagnostics. Nat. Rev. Neurol..

[182] van den Bent M.J., Afra D., de Witte O., Ben Hassel M., Schraub S., Hoang-Xuan K., Malmstrom P.O., Collette L., Pierart M., Mirimanoff R. (2005). Long-term efficacy of early versus delayed radiotherapy for low-grade astrocytoma and oligodendroglioma in adults: The EORTC 22845 randomised trial. Lancet.

[183] Baumert B.G., Hegi M.E., van den Bent M.J., von Deimling A., Gorlia T., Hoang-Xuan K., Brandes A.A., Kantor G., Taphoorn M.J.B., Hassel M.B. (2016). Temozolomide chemotherapy versus radiotherapy in high-risk low-grade glioma (EORTC 22033-26033): A randomised, open-label, phase 3 intergroup study. Lancet Oncol..

[184] Buckner J.C., Shaw E.G., Pugh S.L., Chakravarti A., Gilbert M.R., Barger G.R., Coons S., Ricci P., Bullard D., Brown P.D. (2016). Radiation plus Procarbazine, CCNU, and Vincristine in Low-Grade Glioma. N. Engl. J. Med..

[185] Daniels T.B., Brown P.D., Felten S.J., Wu W., Buckner J.C., Arusell R.M., Curran W.J., Abrams R.A., Schiff D., Shaw E.G. (2011). Validation of EORTC prognostic factors for adults with low-grade glioma: A report using intergroup 86-72-51. Int. J. Radiat. Oncol. Biol. Phys..

[186] Karim A.B., Maat B., Hatlevoll R., Menten J., Rutten E.H., Thomas D.G., Mascarenhas F., Horiot J.C., Parvinen L.M., van Reijn M. (1996). A randomized trial on dose-response in radiation therapy of low-grade cerebral glioma: European Organization for Research and Treatment of Cancer (EORTC) Study 22844. Int. J. Radiat. Oncol. Biol. Phys..

[187] Shaw E., Arusell R., Scheithauer B., O’Fallon J., O’Neill B., Dinapoli R., Nelson D., Earle J., Jones C., Cascino T. (2002). Prospective randomized trial of low- versus high-dose radiation therapy in adults with supratentorial low-grade glioma: Initial report of a North Central Cancer Treatment Group/Radiation Therapy Oncology Group/Eastern Cooperative Oncology Group study. J. Clin. Oncol. Off. J. Am. Soc. Clin. Oncol..

[188] Stupp R., Mason W.P., van den Bent M.J., Weller M., Fisher B., Taphoorn M.J., Belanger K., Brandes A.A., Marosi C., Bogdahn U. (2005). Radiotherapy plus concomitant and adjuvant temozolomide for glioblastoma. N. Engl. J. Med..

[189] Stupp R., Taillibert S., Kanner A.A., Kesari S., Steinberg D.M., Toms S.A., Taylor L.P., Lieberman F., Silvani A., Fink K.L. (2015). Maintenance Therapy With Tumor-Treating Fields Plus Temozolomide vs Temozolomide Alone for Glioblastoma: A Randomized Clinical Trial. JAMA.

[190] Stupp R., Taillibert S., Kanner A., Read W., Steinberg D., Lhermitte B., Toms S., Idbaih A., Ahluwalia M.S., Fink K. (2017). Effect of Tumor-Treating Fields Plus Maintenance Temozolomide vs Maintenance Temozolomide Alone on Survival in Patients With Glioblastoma: A Randomized Clinical Trial. JAMA.

[191] Malmstrom A., Gronberg B.H., Marosi C., Stupp R., Frappaz D., Schultz H., Abacioglu U., Tavelin B., Lhermitte B., Hegi M.E. (2012). Temozolomide versus standard 6-week radiotherapy versus hypofractionated radiotherapy in patients older than 60 years with glioblastoma: The Nordic randomised, phase 3 trial. Lancet Oncol..

[192] Wick W., Platten M., Meisner C., Felsberg J., Tabatabai G., Simon M., Nikkhah G., Papsdorf K., Steinbach J.P., Sabel M. (2012). Temozolomide chemotherapy alone versus radiotherapy alone for malignant astrocytoma in the elderly: The NOA-08 randomised, phase 3 trial. Lancet Oncol..

[193] Bush N.A., Chang S.M., Berger M.S. (2017). Current and future strategies for treatment of glioma. Neurosurg. Rev..

[194] Brem H., Piantadosi S., Burger P.C., Walker M., Selker R., Vick N.A., Black K., Sisti M., Brem S., Mohr G. (1995). Placebo-controlled trial of safety and efficacy of intraoperative controlled delivery by biodegradable polymers of chemotherapy for recurrent gliomas. The Polymer-brain Tumor Treatment Group. Lancet.

[195] Harrington S.E., Smith T.J. (2008). The role of chemotherapy at the end of life:”when is enough, enough?”. JAMA.

[196] McGovern P.C., Lautenbach E., Brennan P.J., Lustig R.A., Fishman N.O. (2003). Risk factors for postcraniotomy surgical site infection after 1,3-bis (2-chloroethyl)-1-nitrosourea (Gliadel) wafer placement. Clin. Infect. Dis.

[197] Stummer W., Meinel T., Ewelt C., Martus P., Jakobs O., Felsberg J., Reifenberger G. (2012). Prospective cohort study of radiotherapy with concomitant and adjuvant temozolomide chemotherapy for glioblastoma patients with no or minimal residual enhancing tumor load after surgery. J. Neuro-Oncology.

[198] McGirt M.J., Than K.D., Weingart J.D., Chaichana K.L., Attenello F.J., Olivi A., Laterra J., Kleinberg L.R., Grossman S.A., Brem H. (2009). Gliadel (BCNU) wafer plus concomitant temozolomide therapy after primary resection of glioblastoma multiforme. J. Neurosurg..

[199] Filley A.C., Dey M. (2017). Dendritic cell based vaccination strategy: An evolving paradigm. J. Neuro-Oncology.

[200] Huang J., Liu F., Liu Z., Tang H., Wu H., Gong Q., Chen J. (2017). Immune Checkpoint in Glioblastoma: Promising and Challenging. Front. Pharm..

[201] Reardon D.A., Brandes A.A., Omuro A., Mulholland P., Lim M., Wick A., Baehring J., Ahluwalia M.S., Roth P., Bähr O. (2020). Effect of Nivolumab vs Bevacizumab in Patients With Recurrent Glioblastoma: The CheckMate 143 Phase 3 Randomized Clinical Trial. JAMA Oncol..

[202] Xiao Q., Yang S., Ding G., Luo M. (2018). Anti-vascular endothelial growth factor in glioblastoma: A systematic review and meta-analysis. Neurol. Sci. Off. J. Ital. Neurol. Soc. Ital. Soc. Clin. Neurophysiol..

[203] Wick W., Weller M., Weiler M., Batchelor T., Yung A.W., Platten M. (2011). Pathway inhibition: Emerging molecular targets for treating glioblastoma. Neuro-Oncology.

[204] Van Den Bent M., Eoli M., Sepulveda J.M., Smits M., Walenkamp A., Frenel J.S., Franceschi E., Clement P.M., Chinot O., De Vos F. (2020). INTELLANCE 2/EORTC 1410 randomized phase II study of Depatux-M alone and with temozolomide vs temozolomide or lomustine in recurrent EGFR amplified glioblastoma. Neuro-Oncology.

[205] Rohle D., Popovici-Muller J., Palaskas N., Turcan S., Grommes C., Campos C., Tsoi J., Clark O., Oldrini B., Komisopoulou E. (2013). An inhibitor of mutant IDH1 delays growth and promotes differentiation of glioma cells. Science.

[206] DiNardo C.D., Stein E.M., de Botton S., Roboz G.J., Altman J.K., Mims A.S., Swords R., Collins R.H., Mannis G.N., Pollyea D.A. (2018). Durable Remissions with Ivosidenib in IDH1-Mutated Relapsed or Refractory AML. N. Engl. J. Med..

[207] Popovici-Muller J., Lemieux R.M., Artin E., Saunders J.O., Salituro F.G., Travins J., Cianchetta G., Cai Z., Zhou D., Cui D. (2018). Discovery of AG-120 (Ivosidenib): A First-in-Class Mutant IDH1 Inhibitor for the Treatment of IDH1 Mutant Cancers. Acs Med. Chem. Lett..

[208] Johannessen T.A., Mukherjee J., Viswanath P., Ohba S., Ronen S.M., Bjerkvig R., Pieper R.O. (2016). Rapid Conversion of Mutant IDH1 from Driver to Passenger in a Model of Human Gliomagenesis. Mol. Cancer Res. Mcr..

[209] Sulkowski P.L., Corso C.D., Robinson N.D., Scanlon S.E., Purshouse K.R., Bai H., Liu Y., Sundaram R.K., Hegan D.C., Fons N.R. (2017). 2-Hydroxyglutarate produced by neomorphic IDH mutations suppresses homologous recombination and induces PARP inhibitor sensitivity. Sci. Transl. Med..

[210] Molenaar R.J., Botman D., Smits M.A., Hira V.V., van Lith S.A., Stap J., Henneman P., Khurshed M., Lenting K., Mul A.N. (2015). Radioprotection of IDH1-Mutated Cancer Cells by the IDH1-Mutant Inhibitor AGI-5198. Cancer Res..

[211] Schumacher T., Bunse L., Pusch S., Sahm F., Wiestler B., Quandt J., Menn O., Osswald M., Oezen I., Ott M. (2014). A vaccine targeting mutant IDH1 induces antitumour immunity. Nature.

